# Targeted degradation of P-TEFb via MDM2 substrate substitution collapses oncogenic transcriptional programs in *MDM2*-amplified cancers

**DOI:** 10.1038/s41419-026-09134-w

**Published:** 2026-08-01

**Authors:** Ye Chen, Long Xie, Xian Guan, Xingze Huang, Jiangyu Liu, Zhipeng Zhu, Yuanfang Wu, Hanjun Guo, Lin Ma, Ying Zhang, Jiawei Zhou, Suya Zheng, Ruhong Zhou, Xin Han, Liang Xu

**Affiliations:** 1 Department of Surgical Oncology, and Pediatric Cancer Research Center, Children’s Hospital, Zhejiang University School of Medicine, National Clinical Research Center for Children and Adolescents’ Health and Diseases, Hangzhou, China; 2 Cancer Center, Zhejiang University, Hangzhou, China; 3 Institute of Biochemistry, College of Life Sciences, Zhejiang University, Hangzhou, China; 4 Cancer Institute (Key Laboratory of Cancer Prevention and Intervention, China National Ministry of Education) of the Second Affiliated Hospital and Institute of Translational Medicine, Zhejiang University School of Medicine, Hangzhou, China; 5 Institute of Biophysics, College of Life Sciences, Zhejiang University, Hangzhou, China; 6 Shanghai Institute for Advanced Study, Zhejiang University, Shanghai, China; 7 Institute of Quantitative Biology, Zhejiang University, Hangzhou, China; 8 Department of Radiation Oncology (Key Laboratory of Cancer Prevention and Intervention, China National Ministry of Education), The Second Affiliated Hospital, Zhejiang University School of Medicine, Hangzhou, China

**Keywords:** Ubiquitin ligases, Drug development

## Abstract

MDM2 is an oncogenic E3 ubiquitin ligase best known for targeting the tumor suppressor p53 and is frequently amplified in human cancers, including dedifferentiated liposarcoma (DDLPS). We show that supraphysiological MDM2 expression can be exploited to reprogram its ligase activity toward selective degradation of oncogenic transcriptional machinery. Using a substrate substitution strategy, we developed an MDM2-recruiting degrader dCDK9-010 that targets positive transcription elongation factor b, inducing its proteasomal degradation while simultaneously stabilizing p53. This dual action disrupts RNA polymerase II abundance and elongation, preferentially impairing enhancer-driven transcriptional programs that sustain tumor growth. In DDLPS models, this approach produces potent antitumor activity with favorable pharmacokinetic and safety profiles, triggers apoptosis, and enhances macrophage-mediated tumor cell clearance. These findings establish substrate substitution-based reprogramming of MDM2 as a generalizable targeted protein degradation strategy and identify *MDM2* amplification as a predictive biomarker for therapeutic response across cancer types, with particular relevance to liposarcoma.

## Introduction

*MDM2* is a well-established oncogene encoding an E3 ubiquitin ligase that degrades p53, the cellular gatekeeper and tumor suppressor [1]. *MDM2* amplification and overexpression occur frequently in human malignancies, marking it a common genetic driver for oncogenesis [2]. The resulting supraphysiologic MDM2 levels in tumor cells create opportunities for precision medicine with MDM2-targeting agents. Agents that disrupt the MDM2-p53 interaction (e.g., Idasanutlin, Siremadlin, and DS-3032) or MDM2 degraders can reactivate p53 to suppress tumor growth [3–8]. Moreover, the E3 ligase activity of MDM2 has been repurposed to degrade non-native substrates (e.g., BRD4 and EZH2) using proteolysis-targeting chimeras (PROTACs) based on small-molecule conjugates [9, 10]. This strategy of redirecting MDM2 for targeted oncoprotein degradation represents a next-generation approach to MDM2-based therapy. However, the repertoire of degradable neo-substrates for MDM2 remains limited. Many attempts to develop MDM2-dependent protein degraders, including those targeting NTRK3 or CDK6, have yielded compounds with minimal or no activities [11, 12], underscoring the need for further research to expand the scope of degradable neo-substrates and advance the development of innovative MDM2-based therapeutic modalities.

Liposarcoma (LPS) ranks among the most common soft-tissue sarcomas, and demonstrates significant molecular heterogeneity with major subtypes including dedifferentiated (DDLPS), well-differentiated (WDLPS), myxoid, pleomorphic, and myxoid pleomorphic LPS. Both WDLPS and DDLPS are molecularly characterized by frequent amplification of chromosome 12q13-15, which drives the overexpression of MDM2 and CDK4 [13]. DDLPS is particularly aggressive, typically presenting as high-grade and therapy-resistant, with high risks of local recurrence and distant metastasis [14]. Consequently, there is an urgent need to develop novel therapeutic strategies for DDLPS. Notably, seminal studies have identified significant transcriptional dependencies in DDLPS pathogenesis, including a disease-specific core transcriptional regulatory circuitry (CRC) driven by super-enhancers through feed-forward transcriptional loops among MYC, RUNX1, and FOSL2 [15–19]. Disruption of this CRC either by direct targeting of its integrity or via bromodomain and extraterminal (BET) protein depletion has demonstrated potent anti-DDLPS efficacy [17], highlighting its essential roles in this disease.

As exemplified by CRC, transcriptional reprogramming is a well-recognized hallmark of cancer, enabling tumor cells to sustain both lineage identity and malignant traits by supporting hyperactive oncogenic mRNA biosynthesis. However, the precise mechanisms through which global RNA Polymerase II (Pol II) levels are adaptively regulated to maintain proficient transcriptional capacity and productive elongation remain incompletely understood. The positive transcription elongation factor b (P-TEFb) complex, a heterodimer of CDK9 and Cyclin T, acts as a central kinase module that phosphorylates Ser2 residues within the heptapeptide repeats of the Rpb1 subunit C-terminal domain (CTD) of Pol II. P-TEFb also interacts with chromatin regulators such as BET bromodomain proteins and the super elongation complex to facilitate transcriptional elongation [20, 21]. Although BET proteins have been shown to preferentially enforce enhancer-driven oncogenic transcription [17], whether P-TEFb exhibits a similar functional bias remains unclear. Beyond its canonical role in promoting productive transcription, P-TEFb, along with other transcription-related kinases, functions to sustain cellular Pol II pools and thereby protects cells from Pol II degradation-dependent apoptotic response (PDAR) [22]. Consequently, the profound transcriptional addiction of tumor cells has been targeted by broad-spectrum transcriptional inhibitors, including SNS032 (targeting CDK2/7/9), Flavopiridol (targeting CDK1/2/4/6/7/9), THZ1 (targeting CDK7/12), Triptolide (targeting transcription factor IIH), and Dactinomycin or α-Amanitin (targeting Pol II), all of which exhibit potent cytotoxic effects. Nevertheless, their clinical utility is substantially hampered by off-target effects, narrow therapeutic windows, and dose-limiting systemic toxicities [23]. As CDK9 is a well-validated oncogenic dependency and therapeutic target [24], the development of novel, highly selective targeting strategies represents a critical next step for successful clinical translation.

The frequent genomic amplification of *MDM2* in human malignancies confers cancer cell-enriched overexpression of its encoded protein. Here, to exploit MDM2’s intrinsic E3 ligase activity for selective degradation of CDK9, we have developed a novel class of “MDM2 substrate substitution-targeting chimeras (MDM2-SubTACs)” that redirect the CDK9/Cyclin T complex to compete for the p53-binding pocket of MDM2, thereby inducing proteasomal degradation of all CDK9 and Cyclin T isoforms. With a focus on *MDM2*-amplified cancers, we systematically investigated the selectivity, mechanism of action, and antitumor potential of our lead compound dCDK9-010, while characterizing the effects of SubTAC-mediated P-TEFb degradation on Pol II abundance, transcriptional elongation, and disease-promoting CRC. MDM2-SubTACs represent an innovative therapeutic modality that leverages tumor-specific MDM2 overexpression, paving the way for next-generation MDM2-targeted therapy.

## Materials and methods

### Cell culture

All cell lines tested negative for mycoplasma and are not listed in the International Cell Line Authentication Committee database. HEK293T (ATCC, Manassas, VA, USA; RRID: CVCL_0063), A172 (ATCC, RRID: CVCL_0131), and MESSA (ATCC; RRID: CVCL_1404) cells were cultured in high-glucose Dulbecco’s Modified Eagle Medium (Meilunbio, Dalian, China). Human adipose-derived mesenchymal stem cells (ADSCs) were obtained commercially and cultured in ncMission hMSC medium (Nuwacell, Hefei, China). DDLPS cell lines LP6 (RRID: CVCL_V413) and LPS141 (RRID: CVCL_M823) were provided by Dr Christopher DM Fletcher (Harvard Medical School). MLS402 (RRID: CVCL_S813), MLS1765 (RRID: CVCL_S817) and GOT3 (RRID: CVCL_M819) cells were generously provided by Dr Pierre Åman (University of Gothenburg). T778 (RRID: CVCL_M808) cells were gifts from Dr Florence Pedeutour (Nice University Hospital). LiSa-2 (LISA2; RRID: CVCL_M821) cells were gifts from Dr Peter Möller (Ulm University). LPS cells were cultured in Roswell Park Memorial Institute 1640 (RPMI-1640) medium (Meilunbio) supplemented with 10% fetal bovine serum (FBS, F101-01; Vazyme, Nanjing, China) and 1% penicillin-streptomycin (ST488S; Beyotime, Shanghai, China). Cells were cultured in a humidified incubator with 5% CO_2_ at 37 °C.

### Key plasmids and chemicals

Target sequences of short hairpin RNAs (shRNAs) and single guide RNAs are listed in Supplementary Table 1. MDM2-3×FLAG and TP53-3×FLAG were inserted into a pCDH-CMV-blast vector modified from pCDH-puro-cMYC (RRID: Addgene_46970). All SNS032-Idasanutlin conjugates were designed and synthesized in-house (Chemical synthesis in Supplementary Methods). Additional key commercially available reagents are listed in Supplementary Table 2.

### Cell lysis and immunoblot assay

Whole-cell extracts were prepared by incubating cells in lysis buffer with fresh addition of protease inhibitors (C0001; TargetMol, Wellesley Hills, MA, USA), phosphatase inhibitors (C0004; TargetMol), 1 mM MgCl_2_, and BenzoNuclease (1:500; M056; Novoprotein, Suzhou, China). Protein concentration was quantified by Bradford assay. Immunoblot analysis was conducted following standard protocol with indicated antibodies listed in Supplementary Table 3. All immunoblots shown are representative of at least two or three independent assays.

### RNA preparation and RNA analysis

Total RNA from cell samples was extracted using FastPure Cell/tissue total RNA isolation kit (RC101-01; Vazyme). Quantitative PCR was performed on a CFX Opus 96 Real-Time PCR System (Bio-Rad, Hercules, CA, USA); primers are listed in Supplementary Table 4. For RNA-seq, RNA integrity was verified prior to library construction. Sequencing libraries were prepared and sequenced on an Illumina platform in paired-end mode. Raw read quality was evaluated with FastQC (v0.12.1) and summarized using MultiQC (v1.15). Reads were aligned to the Ensembl (GRCh38) reference genome, and transcript quantification was performed with STAR (v2.7.11b) and RSEM (v1.3.3), respectively. Differential expression analysis was conducted using DESeq2 (v1.44.0). Gene set enrichment analysis (GSEA) was performed with clusterProfiler (v4.12.6) and the fgsea (1.30.0) packages in R.

### Cleavage under targets and tagmentation (CUT&Tag) assay and analysis

The CUT&Tag assay was performed to map Pol II (Rpb1) genome-wide occupancy in LPS141 cells using the NovoNGS CUT&Tag 4.0 High-Sensitivity Kit (N259-YH01; Novoprotein) following the manufacturer’s protocol. DNA fragments were purified, amplified by PCR, and sequenced on an Illumina platform. Sequencing reads were aligned to the human reference genome (hg38) using Bowtie2 (v2.5.0). Low-quality alignments were filtered with SAMtools (v1.16.1), and high-quality BAM files were normalized to reads per kilobase per million using bamCoverage from deepTools. Peak calling was performed with MACS2 with the parameters (-B -q 0.01 --SPMR). Subsequently, the Pol II pausing indices were calculated as the ratio of Pol II signal density in the promoter regions (TSS ± 500 bp) to that in gene bodies (TSS + 500 bp to TTS + 3 000 bp), providing a quantitative measure of transcriptional pausing and elongation efficiency.

### Molecular docking and ternary complex prediction

Crystal structures of CDK9 (ID: 8K5R) and the p53-binding domain of MDM2 (ID: 4JRG) were retrieved from the Protein Data Bank (PDB). Structures of SNS032-Idasanutlin conjugates were generated from SMILES strings using RDKit (v2023.09.3). The ternary complexes of CDK9–compound–MDM2 (p53-binding domain) were modeled with DeepTernary [25]. Total buried surface area (TBSA) was derived as the sum of buried surface area values across all interaction pairs (i.e., Target–Compound, Target–E3 ligase, and E3 ligase–Compound). Structural alignment of the CDK9/Cyclin T complex and all molecular visualizations were performed in PyMOL (v3.1.0).

### Animal studies

All animal experiments were conducted in accordance with ethical approval from Zhejiang University Institutional Animal Care and Use Committees. For the pharmacokinetic studies, 6- to 8-week-old male CD-1 (ICR, RRID: IMSR_CRL:022; Charles River, Beijing, China) mice were intravenously administered with dCDK9-010 at a dose of 2 mg/kg. Plasma concentrations of dCDK9-010 were quantified by liquid chromatography-tandem mass spectrometry, and pharmacokinetic parameters were determined via non-compartmental analysis using WinNonlin software. To generate subcutaneous xenograft models, 6-to-8-week-old sex-matched NOD/ShiLtJGpt-*Prkdc*^*em26Cd52*^*Il2rg*^*em26Cd22*^/Gpt (NCG) mice (RRID: IMSR_GPT:T001475; GemPharmatech, Nanjing, China) were used as recipients. LPS141 cells were inoculated subcutaneously into the shaved dorsal flank of each mouse. For the pharmacodynamic studies, mice bearing LPS141 xenografts received a single intravenous dose of dCDK9-010 (20 mg/kg). For in vivo efficacy evaluation, once LPS141 xenografts reached a palpable size, mice were randomized into two groups. The treatment group received dCDK9-010 (20 mg/kg, i.v., q.o.d.) over 2 weeks, while the control group received vehicle (5% Solutol HS-15 in PBS) at the same volume and schedule. In the experimental metastasis assay, LPS141 cells were injected via tail vein into the NCG mice. One week after cell inoculation, mice were randomized into treatment and control groups. The treatment group received dCDK9-010 (20 mg/kg, i.v., q.o.d.) over ~3 weeks, while the control group received vehicle on the same schedule. No specific randomization method or blinding was applied.

### Statistical analysis

Unless otherwise stated, statistical significance between two groups was assessed using a two-tailed Student’s t-test for comparisons. Multi-group comparisons were analyzed by the Kruskal–Wallis test followed by Dunn’s test. Animal survival data were analyzed by the log-rank test and visualized with Kaplan–Meier curves. Sample sizes were not pre-determined statistically; the exact sample size (*n*), measure of center (mean or median), measure of dispersion (SEM or SD), and number of independent replicates are reported in each figure legend. Replicates are defined as (i) distinct tumors in xenograft assays, or (ii) independent biological repeats for in vitro assays. For notched box plots in this study: the box bounds represent the 25th and 75th percentiles; the central line indicates the median; the notch shows the 95% confidence interval of the median; whiskers extend to 1.5 times the interquartile range from the box.

## Results

### Harnessing supraphysiological MDM2 for targeted degradation of P-TEFb in DDLPS cells

**Fig. 1:**
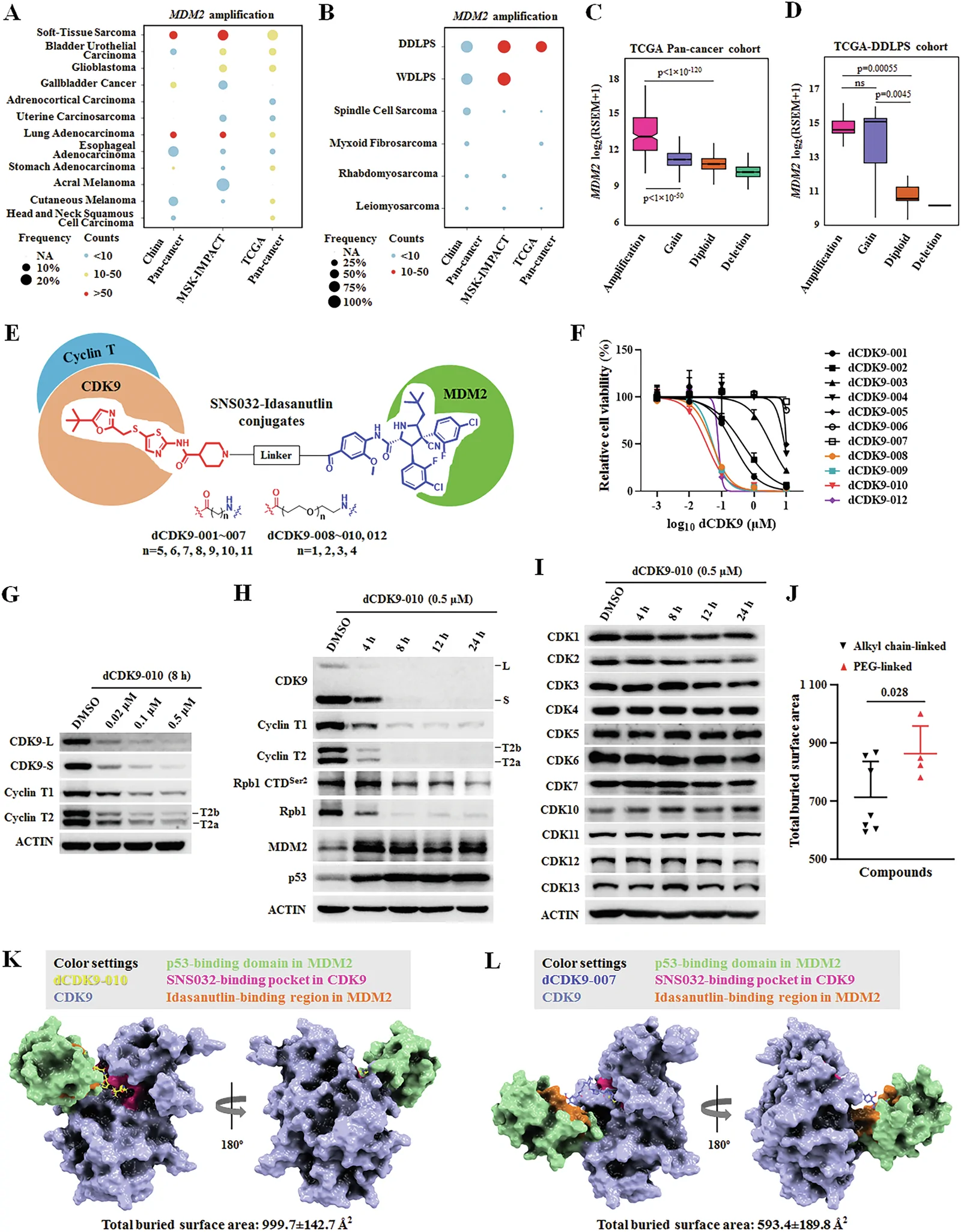
Harnessing supraphysiological MDM2 for targeted P-TEFb degradation. **A**, **B** Recurrent *MDM2* amplification across human cancers and soft-tissue sarcomas across three independent cohorts, including China pan-cancer (*n* = 10 194), MSK-IMPACT (*n* = 10 945), and TCGA pan-cancer (*n* = 10 967). Data were retrieved from the cBioPortal. NA, not available; DDLPS, dedifferentiated liposarcoma; WDLPS, well-differentiated liposarcoma. **C**, **D** Association between *MDM2* copy number variation and transcript levels in pan-cancer and DDLPS cohorts. Gene expression is quantified as RSEM (RNA‑seq by expectation‑maximization). Statistical significance was determined using a post-hoc Dunn’s test after a Kruskal–Wallis test. **E** Design strategy of SNS032-Idasanutlin conjugates for recruiting MDM2 to degrade CDK9 and Cyclin T. **F** Dose–response curves of SNS032-Idasanutlin conjugates in the DDLPS cell line LPS141. **G**–**I** Effects of dCDK9-010 on CDK9, Cyclin T, p53, and other indicated proteins in LPS141 cells. Two isoforms of CDK9 (L and S) and Cyclin T2 (T2a and T2b) are detectable. Rpb1, also known as POLR2A, is the largest subunit of Pol II. **J** Total buried surface area comparison between SNS032-Idasanutlin conjugates with polyethene glycol (PEG) linkers (*n* = 4) and alkyl chain linkers (*n* = 7). Significance was assessed using Student’s *t* test (one-tailed). **K**, **L** Predicted structures of the CDK9–compound–MDM2 (p53-binding domain) ternary complexes modeled with DeepTernary.

To systematically identify *MDM2* amplifications in human cancers, we performed a pan-cancer analysis across three independent cohorts [26–28]. *MDM2* amplification occurs in over 3% of human cancers, but with an exceptionally high frequency in WDLPS and DDLPS (71% ~ 100%), followed by acral melanoma (27%), bladder cancer (7% ~ 9%), esophageal adenocarcinoma (5% ~ 18%), glioblastoma (8% ~ 10%), lung adenocarcinoma (5% ~ 7%), and myxofibrosarcoma (8% ~ 12%) (Fig. 1A, B). *MDM2* amplification is a strong factor positively associated with the elevated *MDM2* transcript levels across these cancers (Fig. 1C, D; Supplementary Fig. 1A, B). Hence, these data demonstrate that amplification drives supraphysiological expression of MDM2, thereby establishing it as a cancer cell-enriched E3 ligase.

To exploit MDM2’s E3 ligase activity for targeted protein degradation of CDK9, we designed heterobifunctional molecules by conjugating the MDM2-binding ligand Idasanutlin to the CDK2/7/9 inhibitor SNS032 via a series of linkers (Fig. 1E). Concurrently, we evaluated the cytotoxic and target-degrading efficacy of the synthesized compounds in DDLPS cells (Table 1). Among these newly synthesized compounds, dCDK9-010 emerges as a lead candidate, demonstrating low nanomolar DC_50_ values against all CDK9 and Cyclin T isoforms and two-digit nanomolar IC_50_ values for cell viability in DDLPS cells (Fig. 1F, G; Supplementary Fig. 2A, B; Table 1). This compound also reduced total and phosphorylated Rpb1 (Rpb1 CTD^Ser2^), while potently stabilizing p53 and MDM2 protein levels with no or only weak effects on other CDK family members within the first 12 hours of treatment (Fig. 1H, I). For several CDKs (e.g., CDK2 and CDK3), which were progressively downregulated by dCDK9-010 after 12 hours, their mRNA levels declined from 8 hours post-treatment (Supplementary Fig. 2C), suggesting a secondary effect of transcriptional perturbation on the abundance of these proteins. Together with the successful degradation of the P-TEFb by SNS032-Idasanutlin conjugates, these data validate CDK9 and Cyclin T as novel and actionable neo-substrates of MDM2.

**Table 1:** Cellular activity profiles of SNS032-Idasanutlin conjugates.

Compound	Linker	DC_50_ (nM)				IC_50_ (nM)		
		CDK9-L	CDK9-S	Cyclin T1	Cyclin T2	LPS141	LP6	ADSCs
dCDK9-001		>500	>500	>500	>500	235.9 ± 53.8	426.4 ± 34.7	>10 000
dCDK9-002		>500	>500	>500	>500	493.5 ± 181.5	3 936.3 ± 386.2	>10 000
dCDK9-003		>500	>500	>500	>500	3 387.0 ± 442.5	4 976.3 ± 84.2	>10 000
dCDK9-004		>500	>500	>500	>500	8 593.0 ± 651.2	9 801.0 ± 375.9	>10 000
dCDK9-005		>500	>500	>500	>500	>10 000	>10 000	>10 000
dCDK9-006		>500	>500	>500	>500	>10 000	>10 000	>10 000
dCDK9-007		>500	>500	>500	>500	>10 000	>10 000	>10 000
dCDK9-008		51.1 ± 17.9	30.5 ± 5.4	47.0 ± 12.7	29.5 ± 10.0	50.8 ± 1.3	333.4 ± 2.3	>10 000
dCDK9-009		18.3 ± 3.9	24.7 ± 3.7	38.0 ± 10.6	7.7 ± 2.1	53.6 ± 1.5	81.9 ± 5.6	>10 000
dCDK9-010		6.5 ± 1.4	6.3 ± 1.0	35.3 ± 7.9	9.2 ± 4.4	34.6 ± 3.7	58.5 ± 1.7	7 254.7 ± 1 424.9
dCDK9-012		11.8 ± 0.87	18.7 ± 2.5	30.9 ± 7.3	11.42 ± 3.9	78.6 ± 0.7	58.5 ± 4.4	1 014.1 + 73.0
Idasanutlin	NA	NA	NA	NA	NA	29.9 ± 0.5	28.3 ± 0.6	3 782.0 ± 190.8
SNS032	NA	NA	NA	NA	NA	98.4 ± 1.4	216.0 ± 21.5	90.2 ± 6.7

*DC*_*50*_ half-maximal degradation concentration (mean ± SD), *IC*_*50*_ half-maximal inhibitory concentration (mean ± SEM). *ADSCs* adipose-derived mesenchymal stem cells. “>500” indicates a DC_50_ exceeding the highest tested concentration (500 nM) in the initial screening; “>10 000” indicates an IC_50_ exceeding the highest tested concentration (10 000 nM) in the initial screening, *NA* not applicable.

Structure-activity relationship analysis revealed that linker composition and hydrophilicity critically determine compound potency (Table 1). Compounds incorporating polyethylene glycol (PEG) linkers generally outperformed those with alkyl chain-based analogs (*p* = 0.003, Fisher’s exact test). Among PEG-linked compounds, dCDK9-010, which incorporates PEG3 exhibited maximal overall activity (Table 1), indicating that linker length also modulates potency. Furthermore, using a deep learning framework DeepTernary [25], we modeled the structures of putative CDK9–compound–MDM2 (p53-binding domain) ternary complexes. PEG-linked compounds with higher degradation efficiency and anti-proliferative activity had larger TBSA values in the predicted ternary complexes than alkyl chain-linked analogs (*p* = 0.028, one-tailed *t* test; Fig. 1J; Supplementary Fig. 3A). For instance, the TBSA inside the ternary complex induced by dCDK9-010 (999.7 ± 142.7 Å^2^), ranked highest among the tested compounds and was markedly larger than that for dCDK9-007 (593.4 ± 189.8 Å^2^) which incorporates an alkyl linker of equivalent length (Supplementary Fig. 3A). Comparative binding-mode analysis revealed substantial differences between the predicted ternary complexes induced by dCDK9-010 and dCDK9-007, particularly at the CDK9–MDM2 and CDK9–SNS032 warhead interfaces (Fig. 1K, L; Supplementary Fig. 3B–E). Consistent with these structural differences, calculated buried surface areas indicate that dCDK9-010 promotes stronger interactions at both interfaces relative to dCDK9-007 (Supplementary Fig. 3F–I). Since buried surface area typically correlates with binding affinity and complex stability, these results suggest that PEG linkers in SNS032-Idasanutlin conjugates favor a conformational alignment that enforces an optimal CDK9–compound–MDM2 ternary complex.

### MDM2-recruiting P-TEFb degraders operate via a substrate substitution mechanism

**Fig. 2:**
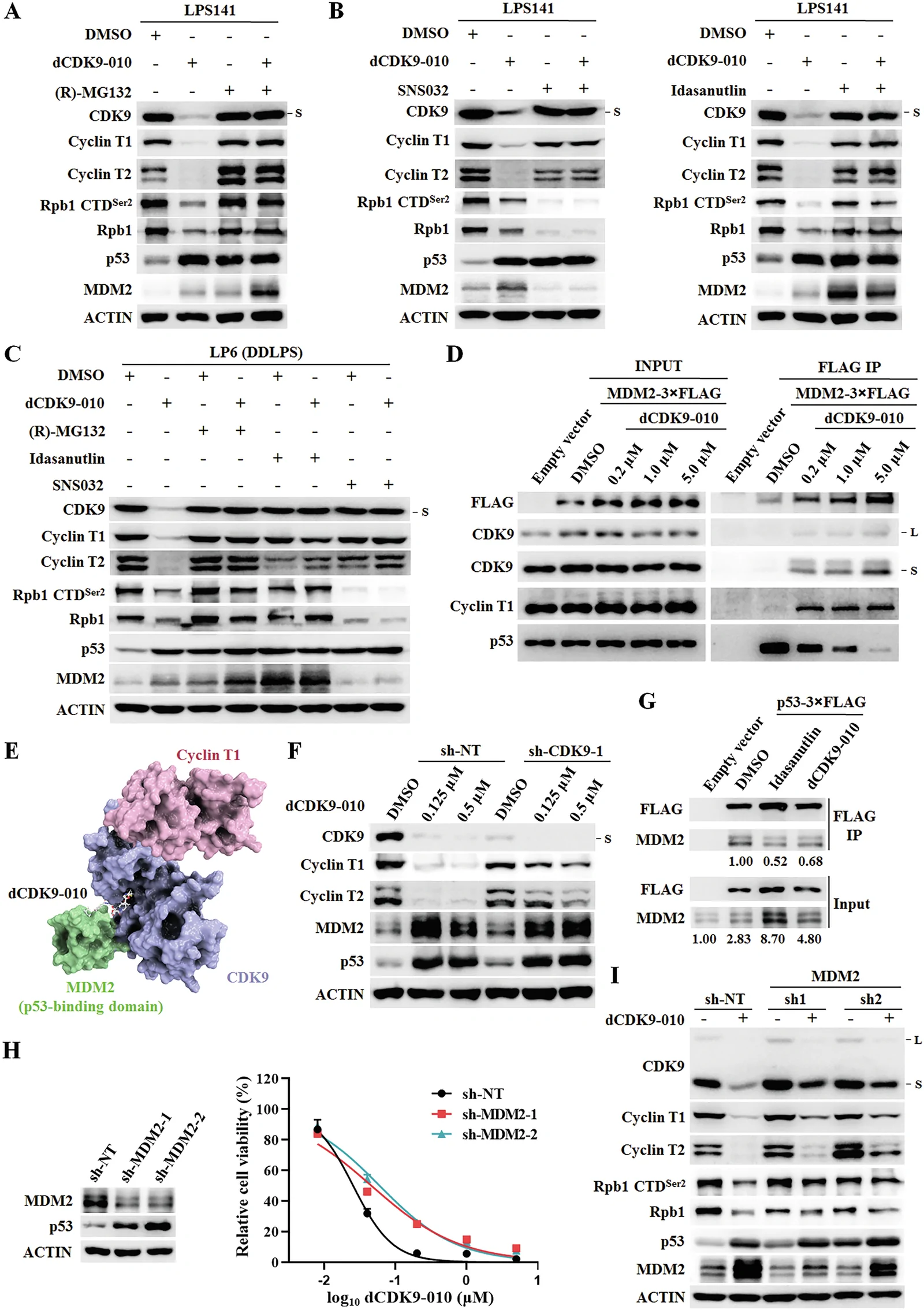
Mechanism of action of the novel MDM2-recruiting P-TEFb degrader dCDK9-010. **A**–**C** Effects of pretreatment with proteasome inhibitor (R)-MG132 (10 μM), the CDK9-binding warhead SNS032 (20 μM), or the MDM2-binding warhead Idasanutlin (20 μM) on dCDK9-010-mediated target protein depletion. DDLPS cells were pretreated for 2 h, followed by 6-hour treatment with 0.5 μM dCDK9-010. **D** dCDK9-010-induced physical interactions among MDM2, CDK9, and Cyclin T1 at the expense of p53 binding. LPS141 cells were treated with indicated concentrations of dCDK9-010 for 1 h, followed by a 2-hour co-incubation with (R)-MG132 (10 μM) before immunoprecipitation. **E** Structural model of the ternary complex formed by P-TEFb (CDK9/Cyclin T1), dCDK9-010, and the p53-binding domain of MDM2. **F** Impact of shRNA-mediated CDK9 knockdown on dCDK9-010-induced Cyclin T degradation. Isogenic LPS141 cells were treated with dCDK9-010 for 8 h. **G** Competitive binding assay showing disruption of the MDM2-p53 interaction by dCDK9-010 (1 μM, 4 h) and Idasanutlin (1 μM, 4 h). **H**, **I** Effects of MDM2 knockdown on dCDK9-010-induced (**H**) cytotoxicity and (**I**) degradation of P-TEFb. Isogenic LPS141 cells were treated with 0.5 μM dCDK9-010 for 8 h.

Given its superior activity in initial screening, dCDK9-010 was selected as the lead compound for mechanistic and functional investigation. We found that the P-TEFb-degrading activity of dCDK9-010 was abolished by proteasome inhibition with (R)-MG132 or by pretreatment with excess ligands (SNS032 or Idasanutlin), indicating a proteasome-dependent and target-engagement-competitive mechanism (Fig. 2A–C). Because dCDK9-010 potently induced endogenous levels of both MDM2 and p53 (Fig. 1H; Supplementary Fig. 4A), we stably expressed either FLAG-MDM2 or FLAG-p53 in LPS141 cells to control for the respective protein levels during subsequent co-immunoprecipitation assays. Surprisingly, dCDK9-010 exerted a positive impact on both the mRNA and protein levels of ectopic FLAG-MDM2 (Supplementary Fig. 4A, B). FLAG immunoprecipitation revealed that dCDK9-010 robustly induced the interaction of exogenous MDM2 with CDK9 and Cyclin T1 while dose-dependently disrupting the MDM2–p53 interaction (Fig. 2D), confirming the formation of a ternary CDK9–compound–MDM2 complex at the expense of p53 binding. Structural alignment of Cyclin T1 with the ternary complex indicated that dCDK9-010 does not disrupt the CDK9–Cyclin T interface (Fig. 2E). Functionally, CDK9 knockdown attenuated dCDK9-010-mediated degradation of both Cyclin T1 and Cyclin T2 (Fig. 2F). Together, these data suggest that SNS032-Idasanutlin conjugates degrade P-TEFb through a hybrid mechanism: the induced MDM2–CDK9 proximity results in CDK9 ubiquitination, and concurrently, CDK9 is exploited as a molecular scaffold to enable the ubiquitination of Cyclin T proteins.

Of note, Idasanutlin was rationally designed to block a specific protein–protein interaction between MDM2 and p53 [29], while most other E3 ligase (e.g., CRBN and DCAF15) ligands act as molecular glues (MGs) that serendipitously induce proximal interactions between neo‑substrate and E3 ligases without disrupting native E3 function [30]. Remarkably, dCDK9-010 preserved Idasanutlin’s capacity to disrupt MDM2–p53 interaction and induce p53 accumulation (Fig. 2G; Supplementary Fig. 4C), unlike MG-based PROTACs, which utilize CRBN- or DCAF15-recruiting ligands (e.g., Thalidomide, Lenalidomide, Indisulam, and E7820) and typically do not interfere with E3’s known native substrates (Supplementary Fig. 4D, E). Hence, our data support a model where the Idasanutlin moiety of dCDK9-010 recruits P-TEFb to MDM2’s p53-binding pocket, leading to p53 displacement and subsequent ubiquitination and proteasomal degradation of P-TEFb. Further supporting this model, silencing MDM2 in DDLPS cells attenuated dCDK9-010 activity (Fig. 2H, I), rendering cells resistant to the compound. Given this unique substrate substitution-based mechanism, we designate this class of PROTAC degraders as MDM2 substrate substitution-targeting chimeras (MDM2-SubTACs).

### MDM2-SubTAC (dCDK9-010) selectively and potently inhibits DDLPS cell viability

**Fig. 3:**
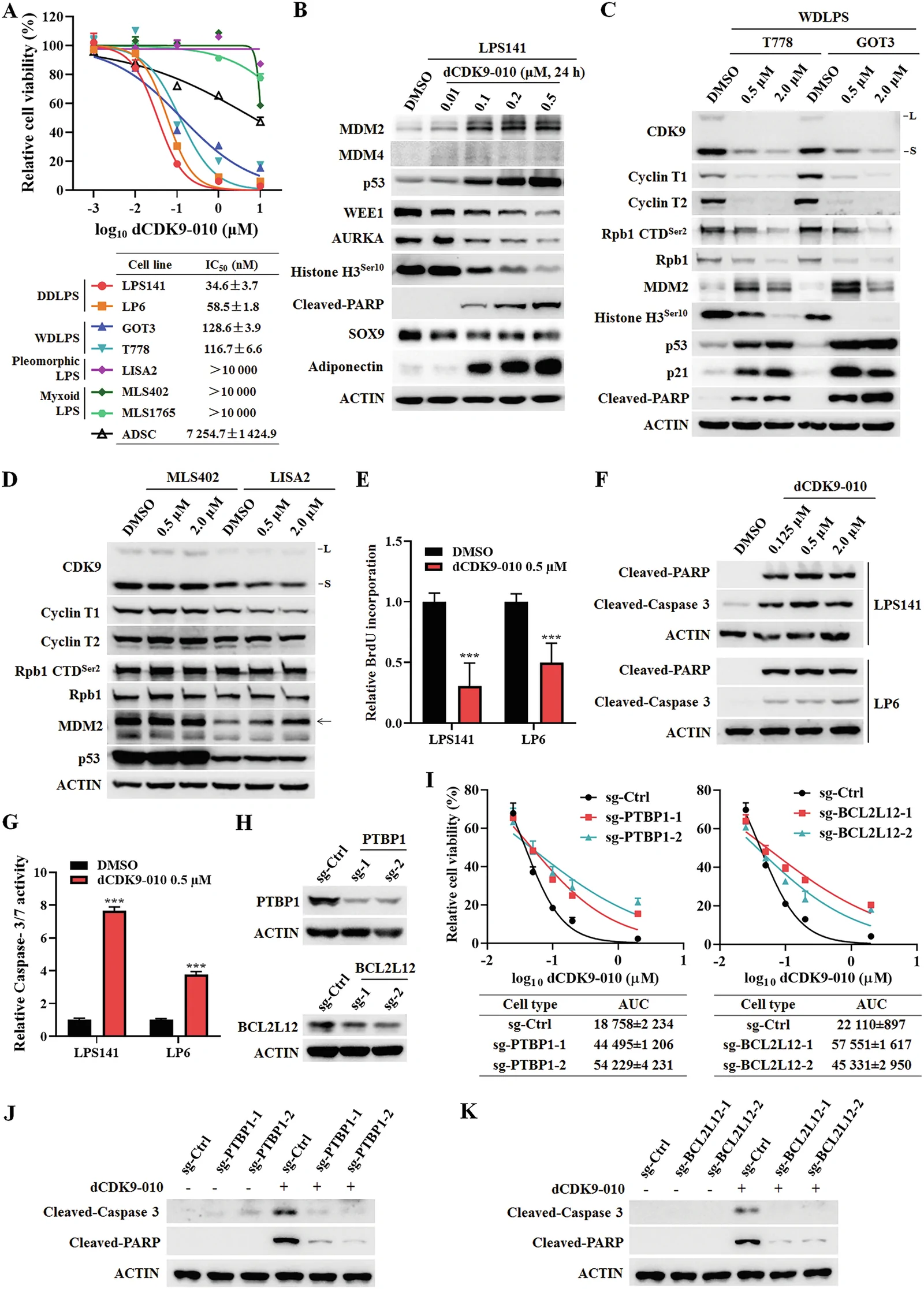
Selective and potent anti-proliferative activity of dCDK9-010 against DDLPS cells. **A** Dose-response curves of dCDK9-010 in LPS cells of various subtypes and adipose-derived mesenchymal stem cells (ADSCs). *TP53*-wild-type, *MDM2*-amplified cell lines: LPS141, LP6, GOT3, T778; *TP53*-wild-type, *MDM2*-diploid cells: ADSCs; *TP53*-wild-type, large T antigen-positive, *MDM2*-diploid cell lines: MLS402, MLS1765; *TP53*-mutated, *MDM2*-diploid cells: LISA2. **B**–**D** Effect of dCDK9-010 treatment (24 h) on indicated proteins in LPS cells of distinct subtypes. Arrow indicates the main MDM2 bands in MLS402 and LISA2 cells. **E** Impact of dCDK9-010 (0.5 μM, 24 h) on BrdU incorporation by DDLPS cells. **F** Effects of dCDK9-010 (24 h) on PARP1 and Caspase 3 cleavage in DDLPS cells. **G** Intracellular Caspase 3/7 activity following dCDK9-010 treatment (0.5 μM, 24 h) in DDLPS cells. **H**–**K** Effects of CRISPR/Cas9-mediated editing of *PTBP1* and *BCL2L12* genes in LPS141 cells on (**H**) intracellular target protein levels, (**I**) cell viability under dCDK9-010 treatment, and (**J**, **K**) dCDK9-010-induced cleavage of PARP1 and Caspase 3. AUC, area under the curve. Data in (**A,** **I**) are presented as mean ± SEM (*n* = 3). Data in (**E**, **G**) are presented as mean ± SD (*n* = 3). Statistical significance in (**E**, **G**) was assessed by Student’s *t* test (two-tailed); ***p < 0.001.

Next, we evaluated the anti-proliferative activity of dCDK9-010 across LPS subtypes and non-malignant cells. We found that dCDK9-010 exhibited potent and selective activity against DDLPS cells, with IC_50_ values ~3- and 100-fold lower than those in WDLPS and other LPS subtypes, respectively (Fig. 3A). In contrast, ADSCs remained largely insensitive to dCDK9-010 (Fig. 3A), highlighting the compound’s exceptional cellular selectivity and establishing *TP53*-wild-type, *MDM2*-amplified DDLPS as a super-responder to this treatment.

In line with its MDM2-dependent mechanism, dCDK9-010 effectively depleted P-TEFb and concomitantly stabilized p53 specifically in DDLPS and WDLPS cell lines harboring *MDM2* amplification (LPS141, T778, GOT3), but not in the myxoid LPS cell line MLS402 (*MDM2*-diploid, *TP53*-wild-type, large T antigen-positive) or pleomorphic LPS cell line LISA2 (*MDM2*-diploid, *TP53*-mutated) (Fig. 3B–D). Accordingly, Rpb1 and p53 levels were responsive to dCDK9-010 treatment in *MDM2*-amplified sarcoma lines but were only minimally affected in *MDM2*-diploid cells harboring either *TP53* mutation or p53 inactivation by large T antigen (Fig. 3B–D). To further reinforce the connection between dCDK9-010 sensitivity and the status of *TP53* and *MDM2*, we examined dCDK9-010 efficacy in the uterine sarcoma cell line MESSA and the astrocytoma cell line A172, both of which harbor wild-type *TP53* and diploid *MDM2*. Coupled with effective P-TEFb depletion and p53 stabilization, dCDK9-010 effectively impaired the viability of these two cell lines, exhibiting an intermediate potency between that observed in WDLPS cells and ADSCs (Supplementary Fig. 5). Together, these data indicate that dCDK9-010 sensitivity correlates with *MDM2* amplification and functional p53, with intermediate effects observed in *TP53*-wild-type and *MDM2*-diploid cells.

Further immunoblot analysis revealed that in responsive LPS141 cells, dCDK9-010 reduced the mitotic marker phospho-Histone H3^Ser10^, key mitotic regulators (AURKA, WEE1), and the dedifferentiation marker SOX9, while elevating the adipogenic differentiation marker Adiponectin, indicative of a shift towards cell cycle exit and differentiation (Fig. 3B). This was accompanied by a significant decline in DNA replication, as shown by 5-Bromo-2’-deoxyuridine (BrdU) incorporation assay (Fig. 3E) and robust induction of apoptosis, evidenced by cleavage of Caspase 3 and PARP1, and elevated Caspase 3/7 activities (Fig. 3F, G). Meanwhile, we also compared the downstream responses following treatment with dCDK9-010, Idasanutlin alone, SNS032 alone, the combination of Idasanutlin and SNS032, and a CRBN-recruiting CDK9 PROTAC (THAL-SNS032), each at the concentration of 0.1 μM. In parallel with dCDK9-010-induced selective depletion of P-TEFb, this MDM2-SubTAC induced either stronger or comparable inhibitory effects on phospho-Histone H3^Ser10^, AURKA, total Rpb1, phospho-Rpb1 CTD^Ser2^, and SOX9 relative to Idasanutlin/SNS032 combination and THAL-SNS032 (Supplementary Fig. 6). Conversely, dCDK9-010 induced the highest level of PARP1 cleavage among all treatment conditions (Supplementary Fig. 6). These observations indicate that dCDK9-010 exhibits both degrader-specific activities (e.g., P-TEFb depletion and SOX9 inhibition) and functional consequences analogous to those of the Idasanutlin/SNS032 combination.

To mechanistically link P-TEFb degradation to cell death, we investigated the recently described Pol II loss-induced apoptotic pathway (known as PDAR) [22]. Genetic ablation of two key PDAR mediators, *PTBP1* and *BCL2L12*, via CRISPR/Cas9 editing significantly enhanced cell survival upon dCDK9-010 treatment, as shown by an increased area under the dose-viability curve (Fig. 3H, I). Consistently, *PTBP1* or *BCL2L12* deficiency markedly attenuated dCDK9-010-induced cleavage of Caspase 3 and PARP1 (Fig. 3J, K). These results demonstrate that PDAR is a critical effector mechanism of dCDK9-010 cytotoxicity.

### MDM2-SubTAC (dCDK9-010) impairs Pol II elongation and oncogenic transcription

Intriguingly, dCDK9-010 consistently reduced both Rpb1 CTD^Ser2^ phosphorylation and total Rpb1 protein levels across all responsive cell lines (Figs. 1H, 2C, 3C). While (R)-MG132 and Idasanutlin fully restored Rpb1 CTD^Ser2^ phosphorylation and total Rpb1 levels, high-dose SNS032 (20 μM) potently suppressed them despite recovering P-TEFb protein (Fig. 2A–C). These results suggest that P‑TEFb integrity and CDK9 kinase activity sustain both Pol II phosphorylation and stability.

**Fig. 4:**
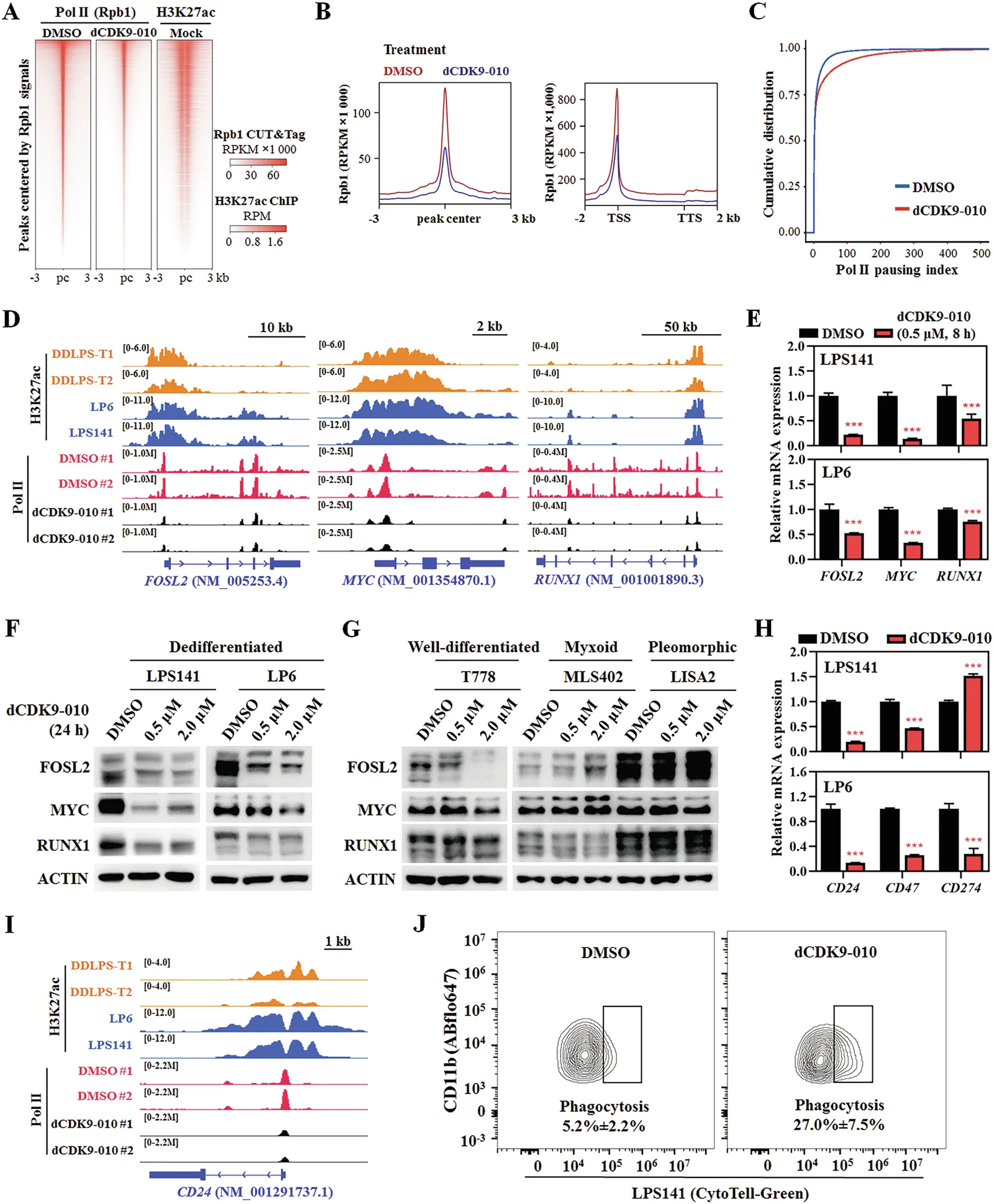
Targeted P-TEFb degradation by dCDK9-010 disrupts Pol II homeostasis and triggers Pol II degradation-dependent apoptotic response. **A** Genomic occupancy of Rpb1 before and after dCDK9-010 treatment (0.5 μM, 8 h). RPKM, reads per kilobase per million mapped reads; RPM, reads per million mapped reads; pc, peak center. **B** Effect of dCDK9-010 treatment on the global Rpb1 occupancy signals and on signals across gene loci. TSS, transcription start site; TTS, transcription termination site. **C** Effect of dCDK9-010 treatment on Pol II pausing indices in LPS141 cells. **D**–**G** Effect of dCDK9-010 on (**D**) Pol II occupancy across the core transcription factor gene loci, (**E**) mRNA and protein levels of these transcription factors in (**F**) DDLPS cells and (**G**) non-DDLPS cells. **H** qPCR analysis of immune checkpoint genes after dCDK9-010 treatment (0.5 μM, 24 h) in DDLPS cells. **I** Effect of dCDK9-010 on Pol II occupancy across the super-enhancer-associated *CD24* in LPS141 cells. **J** Macrophage phagocytosis of LPS141 cells pretreated with dCDK9-010 (0.5 μM, 24 h). Data in (**E**, **H**, **J**) are presented as mean ± SD (*n* = 3). Statistical significance in (**E**, **H**) was accessed by two-tailed Student’s *t* test.

To further characterize the impact of dCDK9-010 on Pol II homeostasis and activity, we performed CUT&Tag-sequencing analysis for Rpb1 in LPS141 cells. In line with the reduction in total Rpb1 levels following P-TEFb degradation, we observed a pronounced decline in global Pol II chromatin occupancy, particularly across gene bodies, after 8-hour compound treatment (Fig. 4A, B). Echoing the downregulation of Rpb1 CTD^Ser2^ phosphorylation by dCDK9-010, Pol II pausing index analysis, which calculates the ratio of promoter-bound versus gene body-bound Pol II signals based on Rpb1 CUT&Tag-sequencing, revealed a substantial impairment of transcriptional elongation across the genome, including super-enhancer-driven oncogenes *RUNX1*, *FOSL2*, and *MYC* (Fig. 4C, D). This resulted in the progressive downregulation of their transcript levels (Fig. 4E). Notably, *RUNX1*, *FOSL2*, and *MYC* have been validated to form a core regulatory circuitry through feed-forward transcriptional amplification that is specifically essential for DDLPS maintenance [17]. In line with both the subtype specificity of this CRC and the cellular selectivity of dCDK9-010, the simultaneous suppression of these core transcription factors was observed exclusively in DDLPS cells, but not in other LPS subtypes (Fig. 4F, G). The selective vulnerability of the DDLPS-specific CRC to P-TEFb degradation likely underlies the disease’s marked hypersensitivity to this MDM2-SubTAC.

Moreover, dCDK9-010 preferentially downregulated the expression of innate immune checkpoint genes (*CD47* and *CD24*) in DDLPS cells, while its effect on the adaptive immune checkpoint *CD274* was variable (Fig. 4H). The downregulation of *CD24* expression was accompanied by a prominent decrease in Pol II loading and travel at its gene locus following dCDK9-010 treatment (Fig. 4I). Concomitantly, dCDK9-010 enhanced macrophage-mediated phagocytosis of DDLPS cells (Fig. 4J), indicating immunomodulatory potential.

**Fig. 5:**
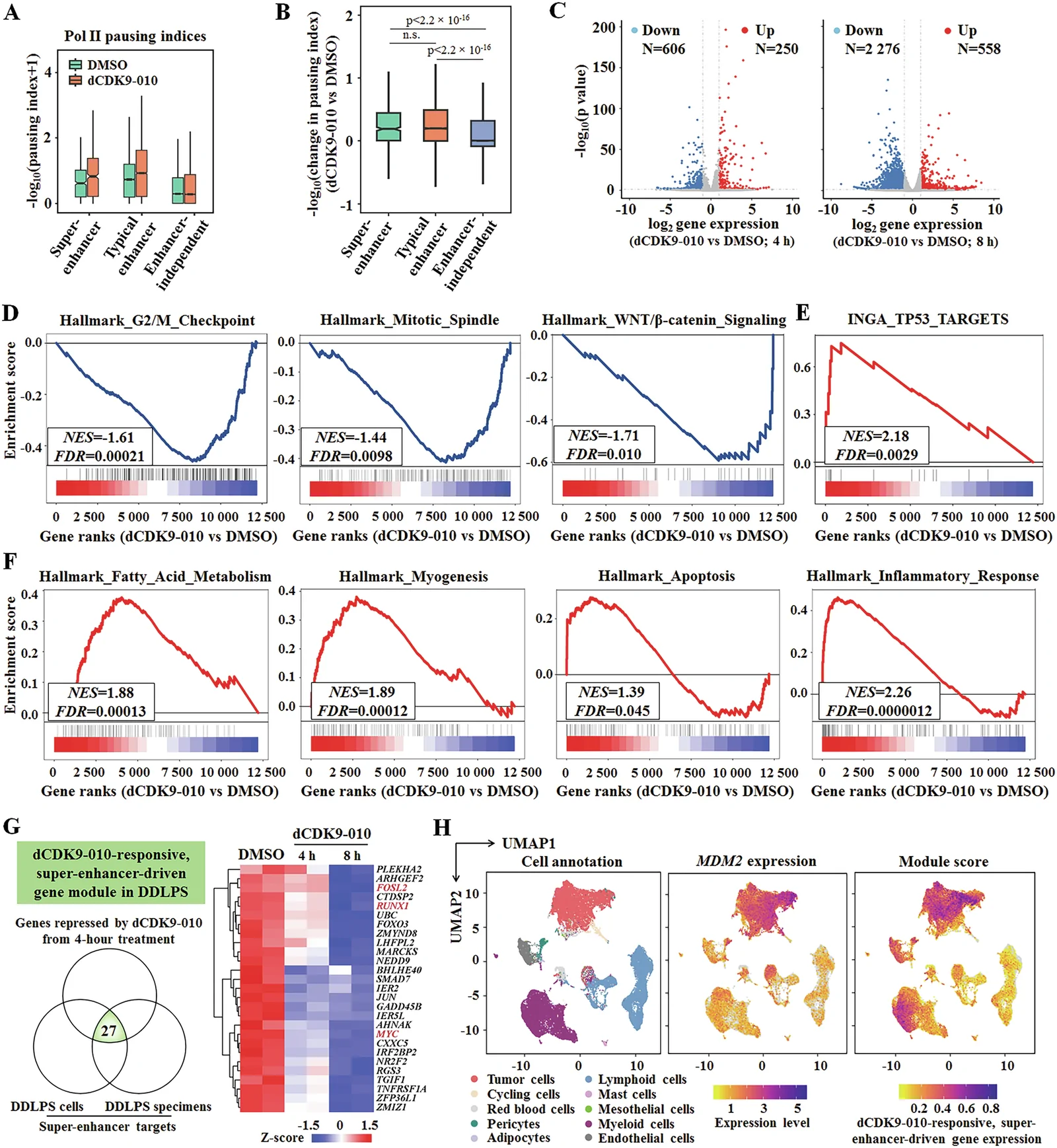
Targeted P-TEFb degradation by dCDK9-010 impairs enhancer-driven transcription and the DDLPS-specific oncogenic program. **A** Effect of dCDK9-010 on Pol II pausing indices for genes associated with different enhancer categories in LPS141 cells. **B** Relative fold changes of Pol II pausing indices for genes associated with different enhancer categories in LPS141 cells upon dCDK9-010 treatment. Statistical significance was determined using a post-hoc Dunn test following a Kruskal–Wallis test; n.s., not significant. **C** Time-course transcriptomic changes in LPS141 cells following treatment with 0.5 μM dCDK9-010. **D**–**F** Representative gene sets (in the human molecular signatures database MSigDB) differentially enriched after dCDK9-010 treatment (0.5 μM, 8 h) in LPS141 cells. NES, normalized enrichment score; FDR, false discovery rate. **G** Identification of a dCDK9-010-responsive, super-enhancer-driven gene module in DDLPS. Super-enhancer-driven gene list is extracted from a previously published work in DDLPS [17]. **H** Single-cell RNA-seq analysis of DDLPS specimens (GSE221493) showing elevated expression of *MDM2* and the dCDK9-010-responsive, super-enhancer-driven gene module in tumor cell population.

Comparative analysis of Pol II pausing indices revealed that under steady-state conditions, super-enhancer-driven genes generally exhibited lower promoter-proximal pausing than typical-enhancer-regulated genes (Fig. 5A). Following dCDK9-010 treatment, however, both categories showed significantly increased Pol II pausing (Fig. 5B). In contrast, enhancer-independent genes remained largely unaffected by dCDK9-010 (Fig. 5A, B), indicating that P-TEFb depletion preferentially disrupts Pol II elongation at enhancer-regulated gene loci.

We next characterized the transcriptional response to dCDK9-010 in DDLPS cells. Time-course RNA-seq analysis uncovered that dCDK9-010 treatment led to predominantly repressive transcriptional changes (Fig. 5C). After 8 hours, dCDK9-010 significantly depleted genes associated with mitotic cell cycle and WNT signaling, while enriching gene sets related to p53 targets, fatty acid metabolism, myogenesis, apoptosis, and inflammatory responses (Fig. 5D–F). Remarkably, a number of super-enhancer-associated genes (including core transcription factors and *JUN*) previously identified in both DDLPS cell lines and clinical specimens were consistently downregulated upon dCDK9-010 treatment (Fig. 5G) [17]. Single-cell RNA-seq analysis of a DDLPS specimen dataset further demonstrated that expression of these dCDK9-010-responsive, super-enhancer-driven genes, as well as *MDM2*, was specifically enriched in the tumor cell population (Fig. 5H; Supplementary Fig. 7). Integrating the MDM2-dependent mechanism of dCDK9-010 with DDLPS cells’ addiction to enhancer-driven transcriptional network, these findings delineate a precision targeting paradigm in which dCDK9-010 exploits tumor-intrinsically enriched MDM2 to degrade P-TEFb, impair Pol II function, and selectively dismantle oncogenic transcriptional homeostasis in DDLPS cells.

While defective P-TEFb has deleterious impacts on transcription, a number of p53 target genes (Fig. 5E) could still be induced by dCDK9-010. To better characterize the tradeoff between global transcriptional inhibition and p53 target induction, we compared the biological effects among dCDK9-010, SNS032, THAL-SNS032, and a well-established Pol II inhibitor Triptolide. Notably, all four agents suppressed the viability of non-malignant ADSCs, with dCDK9-010 demonstrating the most favorable tolerability (Supplementary Fig. 8A). dCDK9-010 and the other transcriptional inhibitors (all at a dose of 0.1 μM for 24 hours) indeed affected general transcription, including uniformly reducing the super-enhancer-associated *JUN* (an oncogenic transcription factor) and *TNFRSF1A* (a p53 target gene) in LPS141 cells (Supplementary Fig. 8B). Importantly, only dCDK9-010 faithfully induced the transcription of *CDKN1A* (encoding p21^Cip1^), in contrast to the repressive effect of Triptolide or the minimal effect of SNS032 and THAL-SNS032 (Supplementary Fig. 8B). Furthermore, immunoblotting results demonstrated that only dCDK9-010 selectively and markedly induced two canonical p53 targets, p21^Cip1^ and PUMA (p53 upregulated modulator of apoptosis), although all treatments except SNS032 elevated p53 protein levels (Supplementary Fig. 8C). Conversely, Triptolide, SNS032, and THAL-SNS032 repressed p21^Cip1^ and PUMA. Among these, Triptolide exhibited the strongest destabilization of Pol II and repression of p53 targets, despite p53 protein induction, suggesting counteractive effects on the transcription of p53 targets. Additionally, consistent with its superior antitumor activity and selectivity, dCDK9-010 exerted relatively stronger effects on inhibiting MYC and inducing cleavage of PARP1 and Caspase 3 than both SNS032 and THAL-SNS032 (Supplementary Fig. 8C). Collectively, these data highlight that dCDK9-010 exhibits a unique mechanism involving P-TEFb depletion and Pol II interference, coupled with persistent activation of p53 and prominent induction of key p53 targets including p21^Cip1^ and PUMA.

### MDM2-SubTAC (dCDK9-010) exhibits potent antitumor efficacy in DDLPS models

**Fig. 6:**
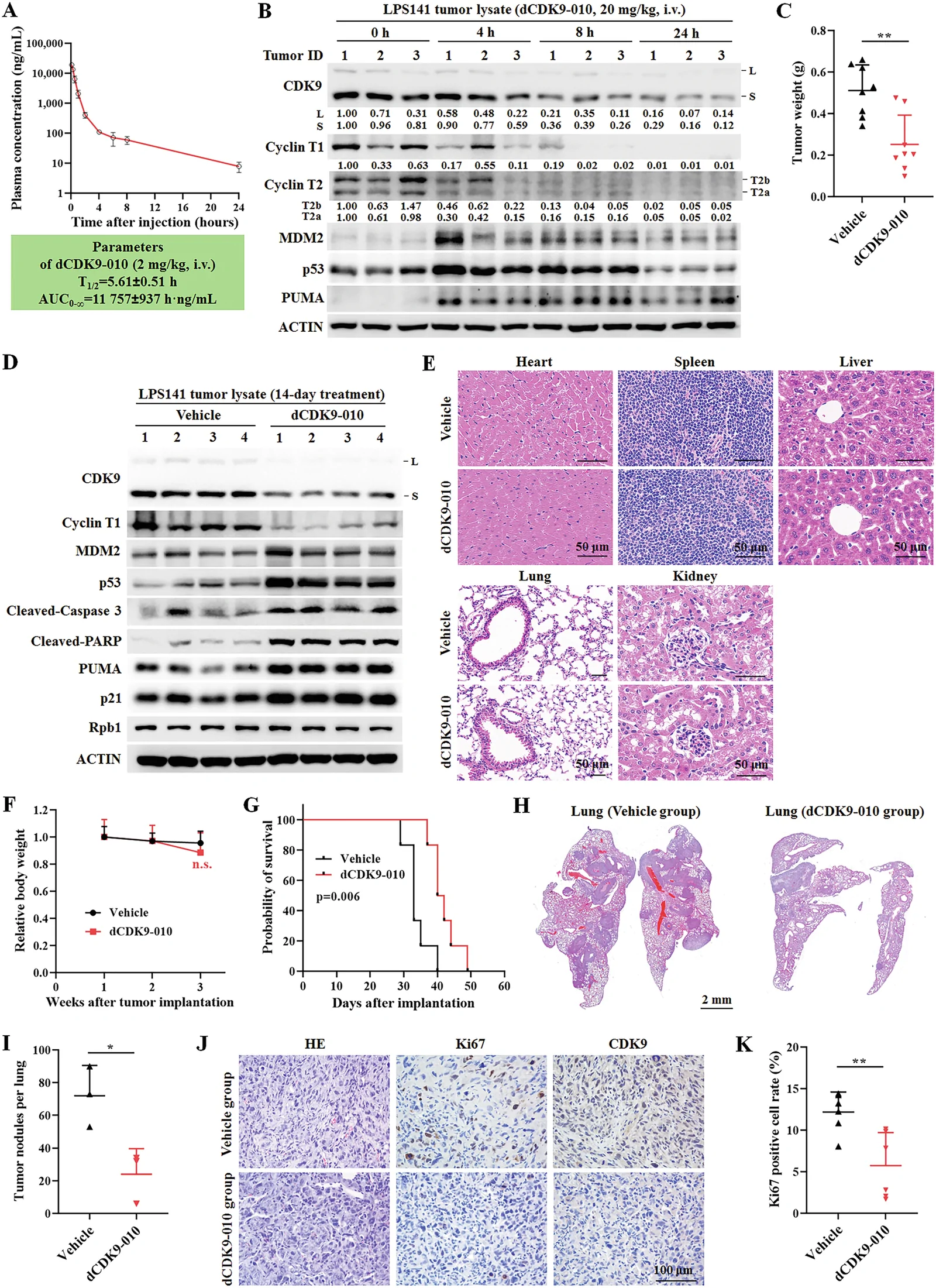
Targeted P-TEFb degradation by dCDK9-010 exhibits potent in vivo activity against DDLPS models. **A** Pharmacokinetics of dCDK9-010 in mice following a single intravenous (i.v.) injection. Data are presented as mean ± SD (*n* = 3). **B** Pharmacodynamic analysis of dCDK9-010 in the LPS141 xenograft model. Immunocompromised mice bearing subcutaneous LPS141 tumors received a single i.v. administration of dCDK9-010 (20 mg/kg) and were euthanized at the indicated time points for tumor collection and immunoblotting. **C** Effects of dCDK9-010 (20 mg/kg, i.v., q.o.d.) on the subcutaneous tumor growth (*n* = 8) of LPS141 cells in immunocompromised mice; significance was assessed by two-tailed Student’s *t* test (**p < 0.01). **D** Expression levels of the indicated proteins in tumor lysates collected at the end of the 14-day treatment with either vehicle or dCDK9-010 (20 mg/kg, i.v., q.o.d.). **E** Histological examination of vital organs from recipient mice after 14-day treatment with either vehicle or dCDK9-010. Scale bar, 50 μm. **F** Effects of dCDK9-010 (20 mg/kg, i.v., q.o.d.) on the mouse body weight. Data are presented as mean ± SD (*n* = 4); significance was assessed by two-tailed Student’s *t* test (**p < 0.01). **G** Effect of 20-day dCDK9-010 treatment (20 mg/kg, i.v., q.o.d.) on overall survival of mice (*n* = 6) bearing distal LPS141 metastases. Survival differences were analyzed by the log-rank test. **H**–**J** Comparative assessment of 20-day dCDK9-010 treatment (20 mg/kg, i.v., q.o.d.) on (**H**, **I**) lung-colonized tumor growth and (**J**) intratumoral expression of Ki67 and CDK9. Data in (**J**) are shown as mean ± SD (*n* = 3); significance was evaluated by two-tailed Student’s *t* test (*p < 0.05). **K** Quantitation of Ki67 expression in lung metastatic xenografts based on immunohistochemical analysis of six representative fields from tumors harvest in (**J**). Statistical significance was assessed by a two-tailed Student’s *t* test.

To evaluate the in vivo activity of dCDK9-010, we investigated its pharmacokinetic profiles in mice and pharmacodynamic effects on DDLPS xenograft models. Intravenous application of dCDK9-010 yielded favorable parameters with a plasma half-life (*T*_1/2_) over 5.5 h (Fig. 6A). Following a single intravenous injection of dCDK9-010 at 20 mg/kg, intratumoral CDK9 and Cyclin T levels were efficiently reduced as early as 4 h post-treatment, and this effect persisted for 24 hours (Fig. 6B). In addition, dCDK9-010 induced the expression of p53, MDM2, p21^Cip1^, and PUMA, whereas progressively downregulating WEE1 and AURKA over the same time course (Fig. 6B; Supplementary Fig. 9A).

The long-lasting (>24 hours) in vivo target degradation efficacy of dCDK9-010 established the rationale for an every-other-day (q.o.d.) dosing schedule to evaluate its antitumor activity in DDLPS xenograft models. Intravenous administration of dCDK9-010 (q.o.d.) over a 14-day treatment period significantly suppressed tumor xenograft growth (Fig. 6C). At the end of the experiment, both xenograft tumors and major organs were harvested for further analysis. Intratumoral protein levels of CDK9 and Cyclin T1 were markedly reduced, whereas levels of p53, MDM2, p21^Cip1^, PUMA, cleaved PARP1, and cleaved Caspase 3 were induced following the 14-day dCDK9-010 treatment (Fig. 6D). In contrast, despite moderate induction of MDM2 levels in murine livers and spleens, protein levels of p53, Rpb1, and CDK9 remained only marginally affected in these normal tissues (Supplementary Fig. 9B). These findings suggest that dCDK9-010 treatment selectively and effectively suppresses DDLPS cell expansion and induces apoptosis to further limit tumor growth. Furthermore, dCDK9-010 treatment neither caused notable damage to major organs nor reduced mouse body weight (Fig. 6E, F). In an experimental metastasis model, dCDK9-010 treatment markedly inhibited lung colonization and outgrowth of LPS141 cells, leading to a significant extension of overall survival of recipient mice (Fig. 6G–I). Immunohistochemical analysis of lung metastases confirmed robust reduction in Ki67 and CDK9 staining signals in the dCDK9-010 treatment group (Fig. 6J, K). Thus, dCDK9-010 exhibited potent anti-DDLPS efficacy and good tolerability in preclinical studies, supporting its further development toward clinical translation.

## Discussion

**Fig. 7:**
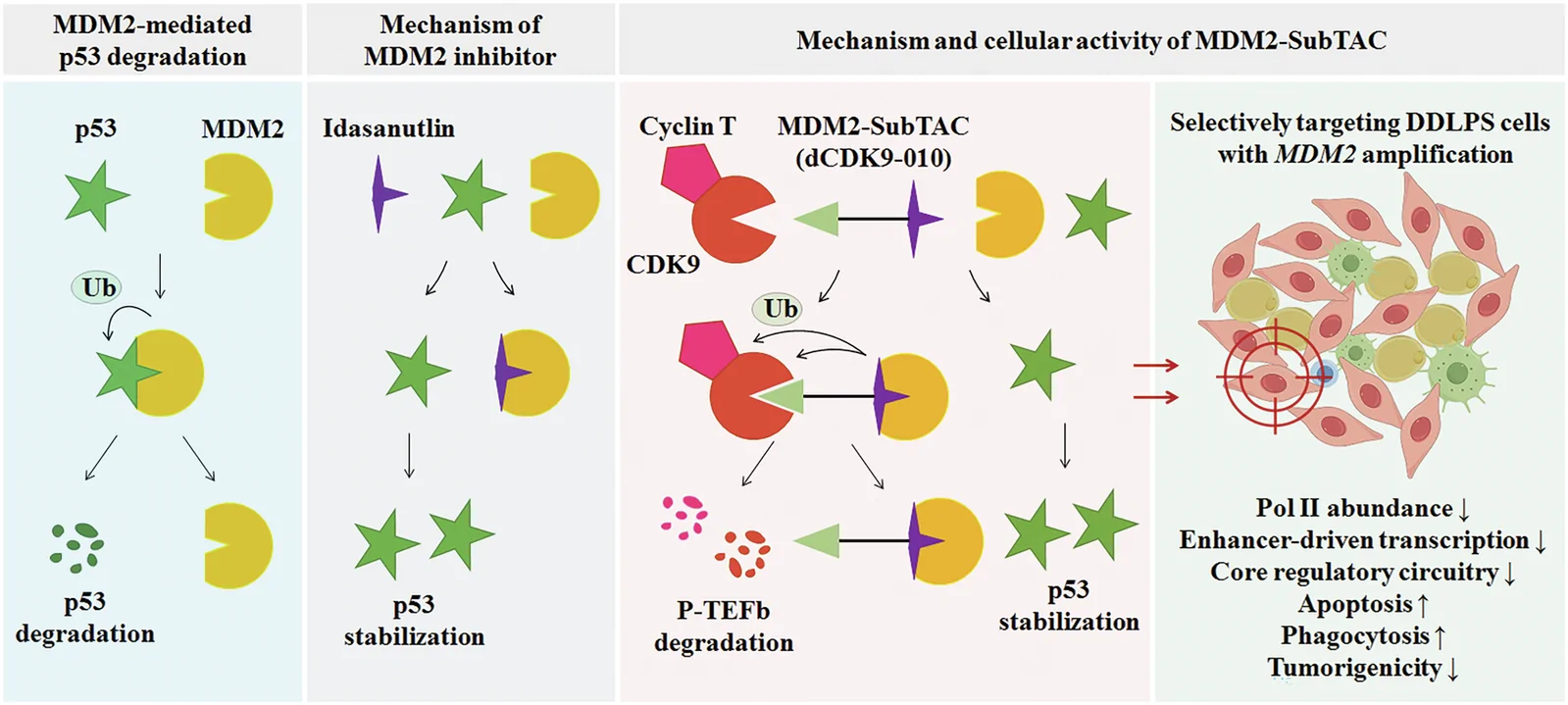
Mechanistic framework of MDM2-SubTAC. Schematic illustration of physiological MDM2 dependent p53 degradation, the mode of action of MDM2 inhibitor, and the MDM2-recruiting SubTAC (dCDK9-010) mechanism, along with its associated cellular activities.

Collectively, our study reports dCDK9-010, a newly developed MDM2-recruiting P-TEFb degrader demonstrating potent and selective activity against DDLPS, accompanied by favorable pharmacokinetic, pharmacodynamic, and safety profiles. Operating through a SubTAC mechanism (Fig. 7), dCDK9-010 not only preserves the p53-stabilizing property of conventional MDM2 inhibitors, but also enables targeted degradation of CDK9 and Cyclin T. This work establishes MDM2-SubTACs as a novel therapeutic strategy that leverages tumor-specific *MDM2* amplification to achieve selective oncoprotein degradation.

Moving beyond conventional ATP-competitive CDK9 inhibitors, we present a proof-of-concept study employing an MDM2 substrate substitution strategy to simultaneously deplete both CDK9 and Cyclin T. To our knowledge, dCDK9-010 represents the first MDM2-recruiting PROTAC capable of degrading all isoforms of CDK9 and Cyclin T. Mechanistically, dCDK9-010 functions through an MDM2 substrate substitution mechanism, which is distinct from most CRBN- or DCAF15-dependent MG-based PROTACs. By retaining Idasanutlin’s ability to occupy the p53-binding pocket of MDM2, dCDK9-010 redirects the E3 ligase toward P-TEFb, resulting in p53 displacement and proximity-induced ubiquitination and degradation of CDK9. Although MDM4 was moderately induced by dCDK9-010 (Fig. 3B), it is likely dispensable for this process, given its very low expression in LPS141 ( < 3 transcripts per million) relative to MDM2 ( > 850 transcripts per million). The concomitant degradation of Cyclin T proteins likely occurs as a bridged effect following CDK9 recruitment to MDM2. An alternative possibility is that MDM2 exhibits greater structural flexibility than other E3 ligases, enabling it to engage and degrade protein complexes such as P-TEFb. Another interesting observation is that dCDK9-010 further elevates the level of ectopically expressed MDM2 (driven by the CMV promoter) even under proteasome blockade, suggesting that it has the potential to regulate MDM2 through mechanisms beyond the well-established p53-dependent feedback transcription.

Compared to existing CDK9 kinase inhibitors [31], CRBN-based CDK9 degraders [32–35], or other degradation strategies such as hydrophobic tagging or autophagy tethering [36–38], dCDK9-010 offers several advantages, including enhanced selectivity for CDK9/Cyclin T, cancer cell-specific activity in *MDM2*-amplified contexts, and favorable pharmacokinetics with robust in vivo efficacy. While dCDK9-010 showed comparable potency to Idasanultin with respect to their effects on LPS141 cell viability, it exerted stronger effects than SNS032 (Table 1). Notably, when compared to SNS032 and Idasanutlin, dCDK9-010 was better tolerated by ADSCs (Table 1), suggesting an improved therapeutic window. Intriguingly, although SNS032 is known to inhibit multiple CDKs, its incorporation into a heterobifunctional PROTAC redirects selectivity from binding to degradation, presumably by altering the efficiency of ternary complex formation and subsequent substrate ubiquitination. Degrading the entire P-TEFb complex offers a potential advantage over CDK9 kinase inhibition by simultaneously targeting its scaffolding functions and the CDK9-independent activities of Cyclin T. Our work not only expands the landscape of MDM2-degradable neo-substrates but also enriches the toolbox for CDK9 disruption and MDM2-based therapeutic intervention.

To date, the development of heterobifunctional protein degraders has remained largely dependent on empirical, trial-and-error screening [39]. Our exploratory study provides valuable insights into artificial intelligence-assisted rational degrader design. Through the employment of a deep learning framework for modeling MDM2–SubTAC–CDK9 ternary complexes, we highlighted the TBSA as a strong candidate structural determinant for predicting degrader efficacy. Within the group of compounds with PEG linkers, TBSA values demonstrated a strong negative correlation with IC_50_ (Spearman *r* *=* −0.8) and a moderate negative correlation with CDK9 DC_50_ (Spearman *r* = −0.4). This parameter integrates contributions from intermolecular ligand–target, ligand–E3 ligase, and target–E3 ligase interactions, thereby serving as a reliable guide for the in silico design, screening, and prioritization of compounds. Intriguingly, other efficacious MDM2-based degraders, such as the BET-targeting A1874 and EZH2-targeting E-3P-MDM2, also employ PEG linkers, suggesting that this linker type may generally favor efficient MDM2-mediated degradation [9,10,40]. Whether PEG linkers generally confer inherent advantages for MDM2-dependent protein degraders remains to be further validated across a broader series of MDM2-SubTACs.

The convergence of amplification-driven supraphysiological MDM2 expression and P-TEFb-dependent transcriptional vulnerability establishes DDLPS as a super-responder to dCDK9-010 among LPS subtypes. This MDM2-SubTAC profoundly impairs DDLPS survival through multiple coordinated mechanisms: disrupting Pol II chromatin loading and elongation, promoting PDAR, collapsing the core oncogenic transcriptional circuitry, activating the p53 tumor suppressor pathway, and enhancing macrophage-mediated phagocytosis of sarcoma cells. While the collapse of the DDLPS-specific transcriptional circuitry is subtype-restricted, the remaining mechanisms are likely applicable to other *MDM2*-amplified cancers.

In vivo studies showed that dCDK9-010 treatment effectively suppressed DDLPS cell proliferation and induced apoptotic markers, but did not induce tumor regression. Combining dCDK9-010 with state-of-the-art cytotoxic agents or immune checkpoint inhibitors may enhance its therapeutic efficacy [41]. Furthermore, the antitumor and immunomodulatory potential of this compound in immunocompetent settings warrants further investigation [42].

The impact of dCDK9-010 on MDM2 induction was evident in DDLPS and WDLPS cells harboring *MDM2* amplification, as well as in MLS402 cells expressing wild-type p53 (whose function is compromised by the large T antigen), but not in *TP53*-mutated LISA2 cells. Despite MDM2 induction, dCDK9-010 failed to degrade P-TEFb in MLS402 cells, suggesting that functional p53 is required for the proteolytic activity of this compound. Additional studies are needed to evaluate the therapeutic potential of dCDK9-010 in non-LPS models with elevated MDM2 or various *TP53* statuses. Nevertheless, as inspired by the current study in LPS, where *MDM2* amplification serves as a predictive biomarker, we propose that MDM2-SubTACs hold promise as tissue-agnostic therapeutics, potentially transforming the treatment landscape for a broad range of *MDM*2-amplified cancers.

## Supplementary information

Supplementary Information (clean version)

Original Experimental Data

## Data Availability

RNA-seq data and CUT&Tag-sequencing data generated in this study have been deposited in the Genome Sequence Archive in National Genomics Data Center, China National Center for Bioinformation (https://ngdc.cncb.ac.cn/gsa) under accession HRA014686. The BED files related to the CUT&Tag-sequencing data have been deposited in the OMIX database (https://ngdc.cncb.ac.cn/omix: accession no. OMIX013849). H3K27ac ChIP-seq data from DDLPS cell lines and primary tissues (GSE111254) and DDLPS single-cell RNA-seq data (GSE221493) are available in the GEO database (https://www.ncbi.nlm.nih.gov/geo/). *MDM2* copy number and the corresponding *MDM2* transcript-level data were retrieved from cBioPortal (https://www.cbioportal.org/) as of 2025-Oct. Correspondence and material requests should be primarily addressed to the lead contact, Dr Liang Xu (xuliang.phd@zju.edu.cn).
