# Supplementary material for: Targeted degradation of P-TEFb via MDM2 substrate substitution collapses oncogenic transcriptional programs in MDM2-amplified cancers
Source: Cell Death Dis. 2026 Aug 1;17(1):920. doi: 10.1038/s41419-026-09134-w (PMC13720051; doi:10.1038/s41419-026-09134-w)
Supplement: Supplementary file 1 [file 41419_2026_9134_MOESM1_ESM.docx]

**Targeted degradation of P-TEFb via MDM2 substrate substitution collapses oncogenic transcriptional programs in *MDM2*-amplified cancers**

By Ye Chen, Long Xie, Xian Guan, Xingze Huang, *et al*.

**Supplementary Information**

- **Supplementary Methods**

Chemical synthesis

Plasmids

Cell lysis and immunoblot assay

Co-immunoprecipitation (Co-IP)

Quantitative reverse transcription polymerase chain reaction (qPCR) analysis

Cell viability assay

BrdU (5-Bromo-2’-deoxyuridine) incorporation assay

Caspase-Glo assay

Animal studies

Hematoxylin and Eosin (HE) staining

Immunohistochemical (IHC) staining

Cleavage under targets and tagmentation (CUT&Tag)

Molecular docking and ternary complex prediction

Single-cell RNA-seq analysis

ChIP-seq analysis

*In vitro* macrophage phagocytosis assay

References

- **Supplementary Figures and Legends**

Supplementary Fig. 1: *MDM2* amplification predicts elevated transcript levels across multiple human cancers.

Supplementary Fig. 2: Activities of SNS032-Idasanutlin conjugates.

Supplementary Fig. 3: Structure-activity relation analysis of SNS032-Idasanutlin conjugates.

Supplementary Fig. 4: Effect of dCDK9-010 on ectopic *MDM2* expression and the effects of E3 ligase-binding ligands on endogenous substrate levels.

Supplementary Fig. 5: Effects of dCDK9-010 treatments on cancer cells with wild-type *TP53* and diploid *MDM2*.

Supplementary Fig. 6: Effects of SNS032, Idasanutlin, SNS032+Idasanutlin combo, dCDK9-010, and THAL-SNS032 treatments (0.1 μM, 24 h) on indicated proteins in LPS141 cells.

Supplementary Fig. 7: Coordinated upregulation of *MDM2* expression and the dCDK9-010-responsive, super-enhancer-driven gene module in the malignant cell compartment of DDLPS.

Supplementary Fig. 8: Comparative study of dCDK9-010 versus established transcriptional inhibitors targeting CDK9 or RNA Pol II.

Supplementary Fig. 9: *In vivo* efficacy analysis of dCDK9-010 in the LPS141 xenograft model.

- **Supplementary Tables**

Supplementary Table 1. Sequences of shRNAs and sgRNAs

Supplementary Table 2. List of commercial chemicals and cytokines

Supplementary Table 3. List of antibodies

Supplementary Table 4. Sequences of qPCR primers

**Supplementary Methods**

**Chemical synthesis.** The SNS032-Idasanutlin conjugates were designed and synthesized in-house. All final compounds were obtained with >95% purity, as determined by ultra-performance liquid chromatography (UPLC). The general synthesis is exemplified by the preparation of dCDK9-010 as below. Briefly, SNS032 was coupled with a linear acid (e.g., 3-(2-(2-(2-(((tert-butoxy)carbonyl)amino)ethoxy)ethoxy)ethoxy)propanoic acid) using 2-(7-azabenzotriazol-1-yl)-N,N,N’,N’-tetramethyluronium hexafluorophosphate (HATU) and N,N-diisopropylethylamine (DIPEA) in N,N-dimethylformamide (DMF) at room temperature. The resulting intermediate was purified and then deprotected with trifluoroacetic acid (TFA)/dichloromethane (DCM‌) to afford the key TFA salt intermediate, 1-(3-(2-(2-(2-aminoethoxy)ethoxy)ethoxy)propanoyl)-N-(5-(((5-tert-butyl-1,3-oxazol-2-yl)methyl)sulfanyl)-1,3-thiazol-2-yl)piperidine-4-carboxamide, in 78% yield over two steps. This intermediate was subsequently coupled with Idasanutlin under similar conditions (HATU/DIPEA, DMF, room temperature). Final purification by preparative high performance liquid chromatography using a C18 column with solvent A (0.1% formic acid in water) and B (0.1% formic acid in methanol) yielded the final compound dCDK9-010, N-(5-(((5-(tert-butyl)oxazol-2-yl)methyl)thio)thiazol-2-yl)-1-(1-(4-((2R,3S,4R,5S)-3-(3-chloro-2-fluorophenyl)-4-(4-chloro-2-fluorophenyl)-4-cyano-5-neopentylpyrrolidine-2-carboxamido)-3-methoxyphenyl)-1-oxo-5,8,11-trioxa-2-azatetradecan-14-oyl)piperidine-4-carboxamide, as a white solid in 55% yield. The structure of the final compound was confirmed by ^1^H nuclear magnetic resonance and ^13^C nuclear magnetic resonance spectra on a Bruker Advance 400 MHz spectrometer, and its purity was assessed by high-resolution mass spectrometry on a Waters UPLC system. Detailed synthetic procedures for other analogs are available upon reasonable request.

**Plasmids**. Short hairpin RNA (shRNA) vectors targeting MDM2 were constructed based on pLKO.1 backbone (RRID: Addgene_8453). Non-target shRNA (SHC002, Sigma-Aldrich, Taufkirchen, Germany) was used as control (sh-Ctrl). All-in-one lentiCRISPRv2-based sgRNA vectors (RRID: Addgene_52961) were constructed to edit *PTBP1* and *BCL2L12*. Target sequences of shRNAs and sgRNAs are listed in Supplementary Table 1. MDM2-3×FLAG and TP53-3×FLAG were inserted to a pCDH-CMV-blast vector modified from pCDH-puro-cMYC (RRID: Addgene_46970). Stable cell lines were generated by lentiviral transduction followed by selection with Puromycin (ST551; Beyotime) or Blasticidin S (ST018; Beyotime).

**Cell lysis and immunoblot assay**. Whole-cell extracts were prepared by incubating cells in lysis buffer (50 mM Tris pH 8.0, 420 mM NaCl, 5% glycerol, 0.1% NP-40, 0.1 mM EDTA) with fresh addition of 1 mM dithiothreitol, 1 mM phenylmethylsulfonyl fluoride (PMSF), 1× protease inhibitor cocktail (C0001; TargetMol, Wellesley Hills, MA, USA), 1× phosphatase inhibitor cocktail (C0004; TargetMol), 1 mM MgCl_2_ and BenzoNuclease (1:500; M056; Novoprotein, Suzhou, China) for 20 min on ice. Clear supernatant after centrifugation (13 000 *g*, 10 min) was collected. Protein concentration was quantified by Bradford assay (P0006C; Beyotime). Immunoblot analysis was conducted following standard protocol with indicated antibodies listed in Supplementary Table 3.

**Co-immunoprecipitation (Co-IP)**. LPS141 cells stably expressing MDM2-3×FLAG were treated with DMSO or dCDK9-010 for 1 h, followed by (R)-MG132 (5 μM) for 2 h before scraping. Cells were lysed in IP buffer (50 mM Tris pH 7.6, 150 mM NaCl, 5% glycerol, 0.1% NP-40, 1 mM EDTA) supplemented with protease and phosphatase inhibitors, 1 mM MgCl_2_, and BenzoNuclease. For each IP reaction, 1 mg of cell lysate was incubated with anti-FLAG M2 affinity gel (A2220; Sigma-Aldrich) at 4℃ for 2 h with gentle rotation. After six washes with IP buffer, bound proteins were eluted and analyzed by sodium dodecyl sulfate-polyacrylamide gel electrophoresis and immunoblotting. Similarly, for substrate competition assays, LPS141 cells stably expressing p53-3×FLAG were treated with indicated compounds for 2 h, then with (R)-MG132 (5 μM) for 2 h prior to harvesting. Cell lysate preparation and immunoprecipitation were performed as described above.

**Quantitative reverse transcription polymerase chain reaction (qPCR) analysis**. Total RNA from cell samples was extracted using FastPure Cell/tissue total RNA isolation kit (RC101-01; Vazyme) according to the manufacturer’s instructions. RNA samples were treated with DNase and subjected to reverse transcription using HiScript III 1st Strand cDNA Synthesis Kit (R312-02; Vazyme). qPCR was performed using ChamQ Universal SYBR qPCR Master Mix (Q711-02; Vazyme) on a CFX Opus 96 Real-Time PCR System (Bio-Rad, Hercules, CA, USA). qPCR primers are listed in Supplementary Table 4.

**Cell viability assay**. Cell viability was measured by an MTT (3-(4,5-dimethylthiazol-2-yl)-2,5-diphenylte-trazolium bromide) colorimetric assay. Cells were plated in 96-well plates at densities ranging from 1 200 to 3 500 cells per well, based on their proliferative capacity. Following 72 h of treatment under indicated conditions, MTT reagent was added to each well and incubated for 3 h. Following the removal of the culture media, MTT STOP solution was added to lyse the cells and solubilize the formazan crystals. The plates were then read on a microplate reader (Molecular Devices, San Jose, CA, USA) with absorbance measured at 570 nm.

**BrdU (5-Bromo-2’-deoxyuridine) incorporation assay**. Sarcoma cells were seeded into a 96-well plate at a density of 5 000 cells per well. After overnight culture, cells were incubated with indicated compounds for another 24 h, followed by addition of BrdU and incubation at 37℃ for 4 h. Cells were then fixed by formaldehyde. BrdU incorporated by proliferating cells was detected by an enzyme-linked immunosorbent assay protocol provided with a BrdU Cell Proliferation Assay Kit (K306; BioVision, Denver, CO, USA).

**Caspase-Glo assay**. Cells were seeded into a white-walled 96-well plate at a density of 3 000-10 000 cells per well. After overnight culture to allow cell attachment, cells were treated with indicated compounds for additional 24 h. A Caspase-Glo 3/7 assay (G8091; Promega, Madison, WI, USA) was conducted according to the manufacturer’s instruction, to monitor the cellular Caspase 3/7 activity which serves as an indicator of apoptosis. The luminescence of each sample was detected by a FlexStation 3 multimode microplate reader (Molecular Devices, San Jose, CA, USA). Culture media without cells were included as a background control.

**Animal studies**. All animal experiments were conducted in accordance with ethical approval from Zhejiang University Institutional Animal Care and Use Committees. For the pharmacokinetic studies, six- to eight-week-old male CD-1 (ICR, RRID: IMSR_CRL:022; Charles River, Beijing, China) mice were intravenously administrated with dCDK9-010 at a dose of 2 mg/kg. Blood samples (approximately 50 µL) were collected from the cheek vein at 5, 15, and 30 min, and at 1, 2, 4, 6, 8, and 24 h post-injection. Plasma concentrations of dCDK9-010 were quantified by liquid chromatography-tandem mass spectrometry, and pharmacokinetic parameters were determined via non-compartmental analysis using WinNonlin software. To generate subcutaneous xenograft models, 6 six- to eight-week-old sex-matched NOD/ShiLtJGpt-*Prkdc^em26Cd52^Il2rg^em26Cd22^*/Gpt (NCG) mice (RRID: IMSR_GPT:T001475; GemPharmatech, Nanjing, China) were used as recipients. LPS141 cells were resuspended in 100 µL of Matrigel (GL101-03; Vazyme)/phosphate-buffered saline (PBS) mixture and inoculated subcutaneously into the shaved dorsal flank of each mouse. For the pharmacodynamic studies, mice bearing LPS141 xenografts received a single intravenous dose of dCDK9-010 (20 mg/kg). Animals were euthanized at 4, 8, and 24 h post-administration, and tumor tissues were harvested for protein extraction and immunoblot analysis. For *in vivo* efficacy evaluation, once LPS141 xenografts reached a palpable size, mice were randomized into two groups. The treatment group received dCDK9-010 (20 mg/kg, i.v., q.o.d.), while the control group received vehicle (5% Solutol HS-15 in PBS) at the same volume and schedule. Tumor dimensions were measured weekly using a caliper to ensure compliance with the institutional ethical limit (maximal diameter: 1.5 cm). At the experimental endpoint, tumors were excised and weighed. In experimental metastasis assay, LPS141 cells (0.15 million per mouse) were injected via tail vein into the NCG mice. One week after cell inoculation, mice were randomized into treatment and control groups. Treatment group received dCDK9-010 (20 mg/kg, i.v., q.o.d.), while the control group received vehicle on the same schedule. Three mice per group were sacrificed two weeks post-treatment for quantification of lung metastatic nodules. The remaining mice were maintained on the treatment regimen, and their survival was monitored on the basis of predefined humane end point criterion (e.g., >20% loss of peak body weight, respiratory distress, or paralysis). No animals were excluded. No specific randomization method or blinding was applied.

**Hematoxylin and Eosin (HE) staining**. Lungs containing metastatic tumors were dissected from mice and fixed in 4% formaldehyde for 24 h. Samples were subsequently embedded in paraffin and sectioned at 5 μm thickness onto adhesive slides (188105W; Citotest, Nanjing, China). After deparaffinization and rehydration through a graded ethanol series, tissue sections were stained with hematoxylin (R20570, Yuanye Bio-Technology, Shanghai, China) for 30 sec at room temperature, followed by a 15-minute rinse under running tap water. Sections were then counterstained with eosin (R20570, Yuanye Bio-Technology) for 30 sec and rinsed again. Sections were finally mounted with neutral mounting medium and imaged using bright-field microscopy.

**Immunohistochemical (IHC) staining**. Paraffin-embedded tumors sections were deparaffinized in xylene and rehydrated through a graded ethanol series. Endogenous peroxidase activity was quenched by incubation in 3% hydrogen peroxide in methanol for 15 min. After antigen retrieval (0.01 M citrate buffer, pH 6.0), sections were incubated with indicated primary antibodies overnight at 4℃, followed by incubation with a horseradish peroxidase-linked secondary antibody. Detection was performed using a DAB^+^ substrate chromogen system (DAKO, Carpinteria, CA, USA). Finally, sections were counterstained with hematoxylin, and mounted. Images were acquired using a high-resolution digital slide scanner.

**Cleavage under targets and tagmentation (CUT&Tag)**. The CUT&Tag assay was performed to map Pol II (Rpb1) genome-wide occupancy in LPS141 cells using the NovoNGS CUT&Tag 4.0 High-Sensitivity Kit (N259-YH01; Novoprotein) following the manufacturer’s protocol. Briefly, 100 000 cells were crosslinked with formaldehyde and harvested for nuclei isolation. The nuclei were then immobilized by Concanavalin A-coated magnetic beads. Following permeabilization, samples were incubated sequentially with anti-Rpb1 antibody (Supplementary Table 3), secondary antibody (A28174; Invitrogen, Waltham, MA, USA), and pAG-Tn5 fusion proteins. MgCl_2_ was added to activate tagmentation, yielding adaptor-ligated DNA fragments. DNA fragments were purified, amplified by PCR, and sequenced on an Illumina platform.

**Molecular docking and ternary complex prediction**. Crystal structures of CDK9 (ID: 8KR5) and the p53-binding domain of MDM2 (ID: 4JRG) were retrieved from the Protein Data Bank (PDB). Structures of SNS032-Idasanutlin conjugates were generated from SMILES strings using RDKit (v2023.09.3). The ternary complexes of CDK9-compound-MDM2 (p53-binding domain) were modeled with DeepTernary (1). The buried surface area was calculated for each interacting pair (i.e., Target-Compound, Target-E3 ligase, and E3 ligase-Compound) within the ternary complex using ChimeraX (v1.9) (2). Total buried surface area was derived as the sum of buried surface area values across all interaction pairs. The following filtering thresholds, which were derived from Target-PROTAC-E3 ligase ternary complexes available from PDB, were applied: buried surface areas between Target-Compound < 506.6 Å^2^; buried surface areas between Target-E3 ligase < 600 Å^2^; buried surface areas between E3 ligase-Compound < 536.3 Å^2^. For each compound, 40 models were generated; only those satisfying all criteria were retained for subsequent analysis. Structural alignment of the CDK9/Cyclin T complex and all molecular visualizations were performed in PyMOL (v3.1.0).

**Single-cell RNA-seq analysis**. Pre-processed scRNA-seq data and associated metadata (dataset GSE221493) were downloaded from the Gene Expression Omnibus (GEO) database and imported into Seurat (v5.2.1) for analysis. Cells derived from DDLPS tissues were extracted for subsequent analysis. Normalization was performed using the SCTransform method, and batch effects were corrected with Harmony (v1.2.3). Dimensionality reduction was carried out via Uniform Manifold Approximation and Projection (UMAP) based on the top 30 principal components. Cell types were annotated according to pre-existing metadata. Scores of super-enhancer target gene list were visualized using AddModuleScore.

**ChIP-seq analysis**. H3K27ac ChIP-seq reads were downloaded from the GEO database (GSE111254) and aligned to the human reference genome (hg38) using BWA-MEM (v0.7.17-r1188; default parameters). Duplicate reads were removed with Picard MarkDuplicates (v2.27.4; --REMOVE_DUPLICATES true) to obtain cleaned BAM files. Predicted fragment length was determined by strand cross-correlation analysis using phantompeakqualtools (v2.0); peak calling was performed using MACS2 (v2.2.7.1, --nomodel). MACS2 (bdgcmp) was used to generate input-subtracted bedGraph tracks, which were converted to bigWig format using the UCSC bedGraphTobigWig utility. Peaks were annotated to genomic features using HOMER (v4.11, annotatePeaks). Super-enhancers and typical enhancers were defined using the ROSE algorithm as previously described (3). Normalized sequencing signals across the selected gene loci were visualized in Integrative Genomics Viewer (v2.14.0). Heatmaps were generated using DeepTools (v3.5.1). Statistical plots were created with ggplot2 (v3.4.4) in R.

***In vitro* macrophage phagocytosis assay**. Adult FVB/NJGpt (RRID: IMSR_GPT:N000026; GemPharmatech) mice were euthanized for bone marrow collection from hind legs. Bone marrow cells were resuspended in macrophage differentiation medium (RPMI-1640 supplemented with 10% FBS, 10 ng/mL M-CSF, and 1% penicillin-streptomycin), and cultured for 6 days with periodic medium refreshment. On day 7, the medium was replaced with M1-polarizing medium (RPMI-1640 supplemented with 10% FBS, 100 ng/mL lipopolysaccharide, 10 ng/mL INF-γ, and 1% penicillin-streptomycin). After 24 h, the differentiated macrophages were dissociated using TryPLE (12563029; Invitrogen), washed with PBS, and resuspended in starvation medium. Concurrently, LPS141 cells pretreated for 24 h with 0.5 μM dCDK9-010 or DMSO were dissociated into single-cell suspensions and stained with CytoTell-Green (22253; AAT Bioquest, Pleasanton, CA, USA). After washing with PBS, the stained LPS141 cells were co-cultured with starved macrophages for 2 h at 37℃. Cells were then resuspended in cell staining buffer (E-CK-A107; Elabscience, Wuhan, China) and incubated with TruStain FcX PLUS (156603; BioLegend, San Diego, CA, USA) for 45 min at 4℃, followed by staining with ABflo647-conjugated anti-mouse CD11b antibody (A24095; Abclonal, Wuhan, China) for 15 min at 4℃. Flow cytometry was performed on a BD LSRFortessa Cell Analyzer (Becton, Dickinson and Company, Franklin Lakes, NJ, USA), and phagocytosis was quantified as the percentage of CD11b^+^ macrophages that were also positive for CytoTell-Green.

**References**

1.	Xue F, Zhang M, Li S, Gao X, Wohlschlegel JA, Huang W, et al. SE(3)-equivariant ternary complex prediction towards target protein degradation. Nat Commun. 2025;16(1):5514.

2.	Pettersen EF, Goddard TD, Huang CC, Meng EC, Couch GS, Croll TI, et al. UCSF ChimeraX: Structure visualization for researchers, educators, and developers. Protein Sci. 2021;30(1):70-82.

3.	Chen Y, Xu L, Mayakonda A, Huang ML, Kanojia D, Tan TZ, et al. Bromodomain and extraterminal proteins foster the core transcriptional regulatory programs and confer vulnerability in liposarcoma. Nat Commun. 2019;10(1):1353.

**Supplementary Figures and Legends**

**Supplementary Fig. 1: *MDM2* amplification predicts elevated transcript levels across multiple human cancers.**

**A**, **B** Association between *MDM2* copy number variation and its transcript levels in (**A**) selected cancer types from the TCGA pan-cancer cohort and (**B**) the TCGA-Sarcoma cohort (liposarcoma excluded). Gene expression is quantified as RSEM (RNA‑seq by expectation‑maximization). For classification, “shallow/deep deletions” are grouped under the “Deletion” category.

**Supplementary Fig. 2: Activities of SNS032-Idasanutlin conjugates.**

**A, B** Assessment of target protein degradation in LPS141 cells treated with distinct SNS032-Idasanutlin conjugates.

**C** Temporal effect of dCDK9-010 treatment (0.5 μM) on the indicated CDK family members in LPS141 cells. Data are presented as mean±SD (n=3). Statistical significance was accessed by one-way ANOVA. ***, p<0.001; **, p<0.01.

**Supplementary Fig. 3: Structure-activity relation analysis of SNS032-Idasanutlin conjugates.**

**A** Total buried surface areas calculated from predicted CDK9-compound-MDM2 (p53-binding domain) ternary complexes. Data are presented as mean±SD from qualified samplings of up to 40 models built by DeepTernary.

**B**, **C** Representative views of predicted ternary complexes showing (**B**) dCDK9-010-mediated and (**C**) dCDK9-007-mediated proximity between CDK9 and MDM2 (p53-binding domain).

**D** Docking poses of Idasanutlin, dCDK9-010, and dCDK9-007 within the drug binding pocket of MDM2 (p53-binding domain).

**E** Docking poses of SNS032, dCDK9-010, and dCDK9-007 within the ATP-binding pocket of CDK9.

**F** Summary of buried surface areas at interaction interfaces for dCDK9-010- and dCDK9-007-induced CDK9-compound-MDM2 (p53-binding domain) ternary complexes. Normally distributed data are shown as mean±SD; non-normal data are presented as median [25%, 75%] from qualified samplings out of 40 predicted models built by DeepTernary.

**G**-**I** Calculated buried surface area at specific interaction interfaces within the CDK9-degrader-MDM2 (p53-binding domain) ternary complex induced by dCDK9-010 and dCDK9-007.

**Supplementary Fig. 4: Effect of dCDK9-010 on ectopic *MDM2* expression and the effects of E3 ligase-binding ligands on endogenous substrate levels.**

**A** qPCR analysis of endogenous and exogenous *MDM2* transcripts following either DMSO or dCDK9-010 treatment. LPS141 cells were treated with indicated concentrations of dCDK9-010 for 1 h, followed by a 2-hour co-incubation with (R)-MG132 (10 μM). One-way ANOVA was applied for statistical analysis.

**B** Examination of dCDK9-010 treatment on the protein levels of MDM2-3xFLAG in LPS141 cells under proteasome blockade. LPS141 cells stably expressing MDM2-3xFLAG were treated with (R)-MG132 (10 μM) for 1 h, followed by incubation with the indicated concentrations of dCDK9-010 for 3 h.

**C-E** Effect of 24-hour treatments on protein levels of known endogenous substrates in LPS141 cells. (**C**) Idasanutlin treatment (MDM2 ligand). (**D**) CRBN ligands (10 μM). (**E**) DCAF15 ligands (10 μM). Protein levels of the respective endogenous substrates for MDM2, CRBN, and DCAF15 were assessed by immunoblotting.

**Supplementary Fig. 5: Effects of dCDK9-010 treatments on cancer cells with wild-type *TP53* and diploid *MDM2*.**

**A** Dose-response curves of cell viability following dCDK9-010 treatment (72 h) in MESSA and A172 cells.

**B** Immunoblotting analysis of target proteins following dCDK9-010 treatment (24 h) in the indicated cell lines.

**Supplementary Fig. 6: Effects of SNS032, Idasanutlin, SNS032+Idasanutlin combo, dCDK9-010, and THAL-SNS032 treatments (0.1 μM, 24 h) on indicated proteins in LPS141 cells.**

**Supplementary Fig. 7: Coordinated upregulation of *MDM2* expression and the dCDK9-010-responsive, super-enhancer-driven gene module in the malignant cell compartment of DDLPS.** Data are from single-cell RNA-seq analysis of DDLPS specimens; significance was determined using a post-hoc Dunn’s test following Kruskal-Wallis analysis.

**Supplementary Fig. 8: Comparative study of dCDK9-010 versus established transcriptional inhibitors targeting CDK9 or RNA Pol II.**

**A** Effects of the indicated agents on the viability of ADSCs and LPS141 cells (72 h). Triptolide, Pol II inhibitor; THAL-SNS032, CRBN-recruiting CDK9 PROTAC.

**B** Effects of the tested agents on the mRNA levels of super-enhancer-associated genes (*JUN*, *TNFRSF1A*) and the well-established p53 target gene *CDKN1A* in LPS141 cells. mRNA levels were measured by qPCR following treatment with each agent (0.1 μM, 24 h). Data are presented as mean±SD (n=3). Statistical significance was accessed by one-way ANOVA; ***, p<0.001; n.s., not significant.

**C** Effects of the tested agents on proteins of interest in LPS141 cells.

**Supplementary Fig. 9: *In vivo* efficacy analysis of dCDK9-010 in the LPS141 xenograft model.**

**A** NCG mice bearing subcutaneous LPS141 xenografts received a single i.v. administration of dCDK9-010 (20 mg/kg) and were euthanized at the indicated time points for tumor collection and immunoblotting.

**B** Expression levels of the indicated proteins in spleen lysates and liver lysates harvested at the end of the 14-day treatment with either vehicle or dCDK9-010 (20 mg/kg, i.v., q.o.d.).

**Supplementary Tables**

**Supplementary Table 1. Sequences of shRNAs and sgRNAs**

| **shRNA** | **Sequence (5’ → 3’)** |
| --- | --- |
| sh-MDM2-1 | CCGGTAGTATAATTGACCTACTTTGCTCGAGCAAAGTAGGTCAATTATACTATTTTTG |
| sh-MDM2-2 | CCGGCTCAGCCATCAACTTCTAGTACTCGAGTACTAGAAGTTGATGGCTGAGTTTTTG |
| sh-CDK9-1 | CCGGAGGGACATGAAGGCTGCTAATCTCGAGATTAGCAGCCTTCATGTCCCTTTTTTG |
|  |  |
| **sgRNA** | **Sequence (5’ → 3’)** |
| sgRNA(#1)-PTBP1 | CACCGGGGCTGGCCGCGCAGCACAG |
| sgRNA(#2)-PTBP1 | CACCGGCTCCCCATCGACGTCACGG |
| sgRNA(#1)-BCL2L12 | CACCGCAGGCTCTAAACCATAGCAG |
| sgRNA(#2)-BCL2L12 | CACCGTGGAGTTGGAACAGGAGACC |

**Supplementary Table 2. List of commercial chemicals and cytokines**

| **Chemical** | **Source** | **Cat #** |
| --- | --- | --- |
| DMSO | Sigma-Aldrich | D4540 |
| Idasanutlin | TargetMol, USA | T6254 |
| SNS032 | TargetMol, USA | T6049 |
| (R)-MG132 | TargetMol, USA | T12628 |
| 7-AAD | Beyotime | ST515 |
| Indisulam | Sigma-Aldrich | SML1225 |
| E7820 | MedChemExpress | HY-14571 |
| Thalidomide | Tocris | 652 |
| Lenalidomide | Selleck | S1029 |
| CytoTell-Green | AAT Bioquest | 22253 |
| Puromycin | Beyotime | ST551 |
| Blasticidin S | Beyotime | ST018 |
| Solutol HS-15 | Aladdin | K487242 |
| Lipopolysaccharide | TargetMol, USA | T11855 |
| M-CSF | Novoprotein | CB34 |
| INF-γ | Novoprotein | C746 |
| Protease inhibitor cocktail | TargetMol, USA | C0001 |
| Phosphatase inhibitor cocktail | TargetMol, USA | C0004 |
| THAL-SNS032 | TargetMol, USA | T17069 |
| Triptolide | Beyotime | Y271881 |

**Supplementary Table 3. List of antibodies**

| **Antibody** | **Brand** | **Cat #** | **RRID** | **Application** |
| --- | --- | --- | --- | --- |
| Adiponectin | HUABIO | HA722286 | AB_3719841 | WB |
| AMPK gamma 1 | Diagbio | db14570 | AB_3717635 | WB |
| AURKA | Cell Signaling Technology | 3092 | AB_2061342 | WB |
| β-ACTIN | Proteintech | 66009-1-Ig | AB_2687938 | WB |
| β-ACTIN | Sigma-Aldrich | A5441 | AB_476744 | WB |
| BCL2L12 | Zenbio | R389049 | AB_3717637 | WB |
| CDK1 | Diagbio | db12527 | AB_3717638 | WB |
| CDK2 | Proteintech | 10122-1-AP | AB_2078556 | WB |
| CDK3 | Uptbio | PLA006035 | AB_3717639 | WB |
| CDK4 | Cell Signaling Technology | 12790 | AB_2631166 | WB |
| CDK5 | Santa Cruz Biotechnology | sc-173 | AB_631224 | WB |
| CDK6 | Santa Cruz Biotechnology | sc-53638 | AB_1121424 | WB |
| CDK7 | Santa Cruz Biotechnology | sc-7344 | AB_627243 | WB |
| CDK9 | Santa Cruz Biotechnology | sc-13130 | AB_627245 | WB, IHC |
| CDK9 | Cell Signaling Technology | 2316 | AB_2291505 | WB |
| CDK10 | Sangon | D220400 | AB 3719840 | WB |
| CDK11 | Cell Signaling Technology | 5524 | AB_10828256 | WB |
| CDK12 | Abclonal | A14894 | AB_2761775 | WB |
| CDK13 | Proteintech | 30461-1-AP | AB_3086325 | WB |
| Cleaved-Caspase 3 | Diagbio | db15982 | AB_3717593 | WB |
| Cleaved-PARP | Cell Signaling Technology | 5625 | AB_10699459 | WB |
| Cyclin T1 | Cell Signaling Technology | 81464 | AB_2799973 | WB |
| Cyclin T2 | Santa Cruz Biotechnology | sc-81243 | AB_1122005 | WB |
| FLAG | Sigma-Aldrich | F1804 | AB_262044 | WB |
| FOSL2 | Cell Signaling Technology | 19967 | AB_2722526 | WB |
| Glutamine Synthetase | Diagbio | db11366 | AB_3717651 | WB |
| Ki67 | DAKO | M7240 | AB_2142367 | IHC |
| MYC | Cell Signaling Technology | 9402 | AB_2151827 | WB |
| MDM2 | GeneTex | GTX100531 | AB_10624270 | WB |
| MDM2 | Diagbio | db12741 | AB_3751485 | WB |
| MDM4 | Santa Cruz Biotechnology | sc-74468 | AB_1126095 | WB |
| ORAI1 | Diagbio | db15735 | AB_3717652 | WB |
| p21 | Cell Signaling Technology | 2947 | AB_823586 | WB |
| p53 | Santa Cruz Biotechnology | sc-126 | AB_628082 | WB |
| p53 | HUABIO | HA722074 | AB_3096714 | WB |
| phospho-Histone H3^Ser10^ | Cell Signaling Technology | 9701 | AB_331535 | WB |
| phospho-Rpb1 CTD^Ser2^ | Cell Signaling Technology | 13499 | AB_2798238 | WB |
| PTBP1 | HUABIO | HA601197 | AB_3071916 | WB |
| PUMA | Santa Cruz Biotechnology | sc-28226 | AB_2064827 | WB |
| RUNX1 | Abcam | ab92336 | AB_2049267 | WB |
| SMC1A | Diagbio | db12036 | AB_3717653 | WB |
| SMC3 | Diagbio | db16147 | AB_3717654 | WB |
| SOX9 | HUABIO | ET1611-56 | AB_2924312 | WB |
| Total-Rbp1 | Diagbio | db15872 | AB_3717592 | WB |
| Total-Rbp1 | Cell Signaling Technology | 2629 | AB_2167468 | WB, CUT&TAG |
| WEE1 | Cell Signaling Technology | 13084 | AB_2713924 | WB |
| ZEB1 | Diagbio | db12557 | AB_3717655 | WB |

**Supplementary Table 4. Sequences of qPCR primers**

| **qPCR primer** | **Sequence (5’ → 3’)** |
| --- | --- |
| ACTB-F | GACGACATGGAGAAAATCTG |
| ACTB-R | ATGATCTGGGTCATCTTCTC |
| CD24-F | CTCCTACCCACGCAGATTTATTC |
| CD24-R | AGAGTGAGACCACGAAGAGAC |
| CD47-F | TATCCTCGCTGTGGTTGGACTG |
| CD47-R | TAGTCCAAGTAATTGTGCTAGAGC |
| CD274-F | ATGCCCCATACAACAAAATC |
| CD274-R | GACATGTCAGTTCATGTTCAG |
| CDK1-F | TTAGCGCGGATCTACCATACCC |
| CDK1-R | GGCTACCACTTGACCTGTAGTT |
| CDK2-F | GTACCTCCCCTGGATGAAGAT |
| CDK2-R | CGAAATCCGCTTGTTAGGGTC |
| CDK3-F | CCAGCTCTTTCGTATCTTTCGT |
| CDK3-R | TTCCTGGTCCACTTAGGGAAG |
| CDK6-F | GGATATGATGTTTCAGCTTCTC |
| CDK6-R | TCTGGAAACTATAGATGCGG |
| CDK7-F | TGTATGGTGTAGGTGTGGACA |
| CDK7-R | TGCAAAGGTATTCCAGGGAAAC |
| CDK12-F | ACCGAAGGGAGAACGACGAA |
| CDK12-R | GAACGCTTTGAACTTCCCGAT |
| CDKN1A-F | TGACAGATTTCTACCACTCCAAACG |
| CDKN1A-R | ATGTAGAGCGGGCCTTTGAG |
| FOSL2-F | AAGAAAAACACCCTGTTTCC |
| FOSL2-R | TATCTACCCGGAATTTCTGC |
| JUN-F | ATCAAGGCGGAGAGGAAGCG |
| JUN-R | TGAGCATGTTGGCCGTGGAC |
| MYC-F | TGAGGAGGAACAAGAAGATG |
| MYC-R | ATCCAGACTCTGACCTTTTG |
| MDM2-F | GAATCATCGGACTCAGGTACATC |
| MDM2-R | TCTGTCTCACTAATTGCTCTCCT |
| Ectopic MDM2-F | GTTTGCCCCTTAATGCCATT |
| Ectopic MDM2-R | CATCGTCATCCTTGTAGTCG |
| RUNX1-F | TTGTGATGCGTATCCCCGTAGA |
| RUNX1-R | GCCGAGTAGTTTTCATCATTGCC |
| TNFRSF1A-F | TCACCGCTTCAGAAAACCACC |
| TNFRSF1A-R | GGTCCACTGTGCAAGAAGAGA |
